# NSrp70 is a lymphocyte-essential splicing factor that controls thymocyte development

**DOI:** 10.1093/nar/gkab389

**Published:** 2021-05-25

**Authors:** Chang-Hyun Kim, Sang-Moo Park, Sun-jae Lee, Young-Dae Kim, Se-Hwan Jang, Seon-Min Woo, Taeg-Kyu Kwon, Zee-Yong Park, Ik-Joo Chung, Hye-Ran Kim, Chang-Duk Jun

**Affiliations:** School of Life Sciences, Gwangju Institute of Science and Technology (GIST), Gwangju 61005, Korea; Immune Synapse and Cell Therapy Research Center, Gwangju Institute of Science and Technology (GIST), Gwangju 61005, Korea; School of Life Sciences, Gwangju Institute of Science and Technology (GIST), Gwangju 61005, Korea; Immune Synapse and Cell Therapy Research Center, Gwangju Institute of Science and Technology (GIST), Gwangju 61005, Korea; School of Life Sciences, Gwangju Institute of Science and Technology (GIST), Gwangju 61005, Korea; School of Life Sciences, Gwangju Institute of Science and Technology (GIST), Gwangju 61005, Korea; Immune Synapse and Cell Therapy Research Center, Gwangju Institute of Science and Technology (GIST), Gwangju 61005, Korea; School of Life Sciences, Gwangju Institute of Science and Technology (GIST), Gwangju 61005, Korea; Department of Immunology, School of Medicine, Keimyung University, Daegu 42601, Korea; Department of Immunology, School of Medicine, Keimyung University, Daegu 42601, Korea; School of Life Sciences, Gwangju Institute of Science and Technology (GIST), Gwangju 61005, Korea; Department of Hematology-Oncology, Immunotherapy Innovation Center, Chonnam National University Medical School, Hwasun 58128, Korea; School of Life Sciences, Gwangju Institute of Science and Technology (GIST), Gwangju 61005, Korea; Immune Synapse and Cell Therapy Research Center, Gwangju Institute of Science and Technology (GIST), Gwangju 61005, Korea; School of Life Sciences, Gwangju Institute of Science and Technology (GIST), Gwangju 61005, Korea; Immune Synapse and Cell Therapy Research Center, Gwangju Institute of Science and Technology (GIST), Gwangju 61005, Korea

## Abstract

Alternative pre-mRNA splicing is a critical step to generate multiple transcripts, thereby dramatically enlarging the proteomic diversity. Thus, a common feature of most alternative splicing factor knockout models is lethality. However, little is known about lineage-specific alternative splicing regulators in a physiological setting. Here, we report that NSrp70 is selectively expressed in developing thymocytes, highest at the double-positive (DP) stage. Global splicing and transcriptional profiling revealed that NSrp70 regulates the cell cycle and survival of thymocytes by controlling the alternative processing of various RNA splicing factors, including the oncogenic splicing factor SRSF1. A conditional-knockout of *Nsrp1* (NSrp70-cKO) using CD4Cre developed severe defects in T cell maturation to single-positive thymocytes, due to insufficient T cell receptor (TCR) signaling and uncontrolled cell growth and death. Mice displayed severe peripheral lymphopenia and could not optimally control tumor growth. This study establishes a model to address the function of lymphoid-lineage-specific alternative splicing factor NSrp70 in a thymic T cell developmental pathway.

## INTRODUCTION

Nuclear speckles are irregular and non-membranous punctuate structures that serve as storage/assembly sites for pre-mRNA splicing machinery, and therefore regulate gene expression (1). Proteins belonging to the nuclear speckles include small nuclear ribonucleoprotein particles (snRNPs) and many non-snRNP splicing factors such as SR (serine/arginine) proteins and heterogeneous nuclear ribonucleoproteins (hnRNPs) (2). Previously, our laboratory discovered a new SR-related protein named nuclear speckle-related protein 70 (NSrp70), which co-localizes and interacts physically with SRSF1 and SRSF2 in nuclear speckles through its RS-like region. NSrp70 modulates alternative splicing site selection of some mRNAs, including Fas v6, Tra2β1 v2 and CD44 exon v5 (3). However, little is known about the significance of NSrp70 in a physiological setting. One clue is that, like other major splicing factors, a knockout of NSrp70 resulted in an embryonic lethal phenotype (3), suggesting that this protein is essential for early development. Interestingly, NSrp70 is predominantly expressed in immune function-related tissues or organs (3), suggesting its potential significance in the immune system. This fact led us to investigate whether NSrp70 plays a role as a lineage-selective splicing regulator in the immune system.

The immune system is where rapid cell division and complex developmental processes occur during hematopoiesis. Notably, T cell development in the thymus is a highly integrated process with distinct developmental stages defined by the expression of the co-receptors CD4 and CD8 (4). These events are primarily driven by the expression and activation of the T cell receptor (TCR) complex. In particular, precise control of TCR signaling is critical for complete thymocyte development (5–7). Further, many factors that regulate the cell cycle, apoptosis, survival, differentiation, and proliferation are also cooperatively involved in thymocyte development (8–11).

A previous report demonstrated that conditional deletion of the prototypical SR protein SRSF2 (also known as SC35) in the thymus causes a defect in T cell maturation (12). An hnRNP L knockout resulted in decreased thymic cellularity caused by a partial block at the transition between DN4 and DP thymocytes and higher proliferation rates in DN4 thymocytes (13). However, the actions of SRSF2 and hnRNP L are not restricted to lymphocytes; indeed, they impact almost all biological processes (14). To our surprise, NSrp70 was highly and selectively detected in developing lymphocytes—highest in DP thymocytes—, strongly suggesting that NSrp70 may have lymphocyte-selective activity during the developmental process.

To address a potential role of NSrp70 in T cell development, we created a conditional-knockout using the CD4Cre-loxP strategy. NSrp70 deletion (*Nsrp1^f/f^*CD4Cre) profoundly perturbed the late development of DP thymocytes, leading to a significant reduction of CD4^+^ and CD8^+^ single positive (SP) cells in the thymus and peripheral lymphoid tissues. Global splicing profiling revealed that NSrp70 controls the processing of cell cycle-related RNA splicing factors, including *Srsf1, Srsf7* and *Son* (15–17), and spliceosome-related factors, including *Sf3b1* and *U2af2* (18). NSrp70 also organizes the nuclear speckles by physically integrating the RNA splicing proteins and governs the cell cycle and survival at the stage of TCR-mediated positive selection to produce CD4^+^ or CD8^+^ SP T cell populations.

## MATERIALS AND METHODS

### Reagents and antibodies

Antibodies against NSrp70 (HPA015603) and mTCRβ (MABF931), tetramethylrhodamine (TRITC)-phalloidin, poly-l-lysine (PLL), A23187, polybrene, and phorbol 12-myristate 13-acetate (PMA) were purchased from Sigma-Aldrich (St. Louis, MO, USA). Anti-TCR ζ-chain (ab40804) antibody was purchased from Abcam (Cambridge, MA, USA). Antibodies against PLCγ1 (sc-7290), phospho-Vav1 (sc-16408-R), SON (NREBP, sc-398508), HDAC1 (10E2), SRSF1 (SF2/ASF, sc-33652) were purchased from Santa Cruz Biotechnology (Dallas, TX, USA). Antibodies against phospho-TCR ζ-chain (PA5-37512), CD45ALL (30-F11), CD45RB and mCherry (PA5-34974) and Fluo-3/AM were purchased from Invitrogen (Carlsbad, CA, USA). Antibodies against β-actin (8457), Zap70 (2705S), phospho-Zap70 (2701S), phospho-PLCγ1 (2821L), PKCθ (12206S), phospho-PKCθ (9376), Erk1/2 (9102S), phospho-Erk1/2 (9101S), p38 kinase (9212S), phospho-p38 kinase (9215S), rabbit IgG (7074) and mouse IgG (7076) were purchased from Cell Signaling Technology (Danvers, MA, USA). Anti-CD2 antibody (130-100-617) was purchased from Miltenyi Biotec (Bergisch Gladbach, Germany). 4′,6-Diamidino-2-phenylindole dihydrochloride (DAPI) was purchased from Molecular Probes (Eugene, OR, USA). Anti-CD28 (ab205136) antibody was purchased from BD Biosciences (San Jose, CA, USA). Hybridoma cell lines for anti-CD3ϵ (145-2C11; CRL-1975), and anti-CD28 (PV1; HB-12352) were purchased from the American Type Culture Collection (Manassas, VA, USA). Antibodies for fluorescein isothiocyanate (FITC)-conjugated mCD4, CD62L, Foxp-3, peridinin chlorophyll protein complex (PerCP) Cy5.5-conjugated CD8α, CD44, TCRβ and for phycoerythrin (PE)-conjugated mouse CD25, CD24, CD69, CXCR4, CCR7, CD3ϵ and TCR ζ, and for PE-Cy7-conjugated mouse CD127 (IL7Rα), CD69 and Bcl-2 were purchased from eBioscience (San Diego, CA, USA). Total RNA isolation reagent was purchased from Molecular Research Center, Inc. (Cincinnati, OH, USA). PCR premix and restriction enzyme were purchased from Enzynomics (Daejeon, Korea). Plasmid DNA purification kit and WEST-ZOL western blot detection kit were purchased from Intron Biotechnology (Seongnam, Korea). PrimeSTAR HS DNA polymerase was purchased from TaKaRa Bio (Shiga, Japan). Magna RIPTM Kit was purchased from EMD Millipore (Billerica, MA, USA). All cell culture materials used in this work were from Life Technologies (Carlsbad, CA, USA). Tissue-Tek OCT was purchased from Sakura Finetek (Torrance, CA, USA). Complete protease inhibitors and phosphatase inhibitors were purchased from Roche Applied Science (Indianapolis, IN, USA). Aqua C18 (particle size 5 μm) reverse phase column material was purchased from Phenomenex (Torrance, CA, USA). Strong cation exchange (particle size 5 μm) column material was purchased from Whatman (Maidstone, Germany).

### Biological resources

#### Cells

Mouse thymocytes were purified from the mouse thymus. Mouse splenocytes and lymphocytes were dispersed and purified to CD4^+^, CD8^+^, CD19^+^ and CD11c^+^ populations by MACS cell separation (Miltenyi Biotec). Naïve CD3^+^ T cells were purified from the mouse spleen and lymph nodes by negative selection using a T cell enrichment column (R&D Systems). To generate mouse T cell blasts, naïve CD3^+^ T cells were incubated in 2 μg/ml anti-CD3/28-coated culture plates with 100 U/ml rIL-2 for 48 h and cultured for a further 5 days with 100 U/ml rIL-2. Mouse splenocytes were dispersed and purified into CD4^+^, CD8^+^ and CD19^+^ populations using the EasySep magnetic separation system (Stemcell Technologies, Vancouver, Canada) or MACS cell separation (Miltenyi Biotec, Bergisch Gladbach, Germany). The purity of each population was confirmed as >95% by flow cytometry. HEK293T cells (CRL-1573, ATCC), B16F10 cells (CRL-6475, ATCC) and EO771 cells (CRL-3461, ATCC) were maintained in DMEM supplemented with 10% FBS, penicillin and streptomycin.

#### Constructs

The mammalian expression vector of the Fas minigene was a gift from Dr Michael Sattler (GSF-National Research Center for Environment and Health, Neuherberg, Germany). pEGFP-C1_NSrp70, and pEGFP-C1_RS1M constructs were generated using a previously reported method (3,19). The cDNA of SRSF1, U2AF1, hnRNP U, U170K was obtained by RT-PCR using total RNA prepared from Jurkat T or HEK293T cells as a template. Then, each cDNA was inserted into the pCS4-3Myc, pmCHerry-C1 and pHJ-1 vectors by in-frame fusion.

#### Animals

For generation of NSrp70-cKO mice, we used the gene-targeting vector with which the two-*loxP* and two-*frt* strategy was employed to obtain homologous recombination in embryonic stem (ES) cells. After successful germ line transmission (Macrogen, Korea), chimera mice (*Nsrp1^+^*^/flox-frt-neo^) were generated, backcrossed onto C57BL/6 background for six generations, and the off-spring were interbred. The *Nsrp1^+^*^/flox-frt-neo^ mice were crossed with β-actin-driven *Saccharomyces cerevisiae*-enhanced FLP1 recombinase variant (FLPe) deleter mice (The Jackson Laboratory) to facilitate *in vivo frt-neo* deletion. The *Nsrp1*^flox/flox^ (*Nsrp1*^f/f^ will be used herein to indicate *Nsrp1*^flox/flox^ for simplicity) mice were further crossed with CD4cre transgenic mice (The Jackson Laboratory) to generate *Nsrp1*^f/f^CD4cre mice. All mice were housed in specific pathogen-free conditions. All experimental methods and protocols were approved by the Institutional Animal Care and Use Committee of the School of Life Sciences, Gwangju Institute of Science and Technology (Gwangju, Korea), and carried out in accordance with their approved guidelines.

### Cell transfection and lentiviral infection

HEK293T cells were transfected with various constructs (pHJ-1_NSrp70, and pHJ-1_SRSF1) by Lipofectamine 2000 (Life Technologies) according to the manufacturer's instructions. To establish stable cell lines, the pHJ-1 vector was co-transfected with lentiviral packaging vectors into HEK293T cells. After 48 h, the supernatants were collected and spin-infected into HEK293T cells by centrifugation at 800 × g for 90 min in the presence of polybrene (8 μg/ml).

### Western blot analysis

Cells or homogenized tissues from a C57BL/6 mouse were lysed in ice-cold lysis buffer (50 mM Tris–HCl pH 7.4, containing 150 mM NaCl, 1% Triton X-100, one tablet of complete protease inhibitors and phosphatase inhibitors) for 30 min on ice. Cell lysates were centrifuged at 16 000 × *g* for 25 min at 4°C, and the supernatant was eluted with SDS sample buffer (100 mM Tris–HCl pH 6.8, 4% SDS and 20% glycerol with bromophenol blue) and boiled for 5 min. The proteins were separated through 10–12% SDS-PAGE gels and were transferred to a nitrocellulose membrane by means of a Trans-Blot SD semidry transfer cell (Bio-Rad, California, USA). The membrane was blocked in 5% skim milk for 1 h, rinsed, and incubated with intended antibodies in TBS containing 0.1% Tween 20 (TBS-T) and 3% skim milk overnight. Excess primary antibody was then removed by washing the membrane four times in TBS-T. The membrane was then incubated with peroxidase-labeled secondary antibody (0.1 μg/ml, anti-rabbit or -mouse) for 1 h. After three washes with TBS-T, bands were visualized by WEST-ZOL western blot detection kit (Intron Biotechnology) and the membranes were then exposed to X-ray film.

### Immunoprecipitation (IP)

Cell lysates were precleared, and supernatants were incubated overnight with antibodies at 4°C, followed by incubation with protein A/G agarose beads (Santa Cruz Biotechnology). Beads were collected, washed with PBS, and resuspended in equal volumes of 5× SDS loading buffer. Immunoprecipitated proteins were separated by SDS-PAGE on 10, and 12% gels and analyzed by western blot analysis as described above.

### mRNA-seq and data analysis

Total RNA was purified from sorted-DP thymocytes using TRIzol reagents (Molecular Research Center, Cincinnati, OH, USA). A NanoDrop spectrophotometer (Thermo Fisher Scientific Inc., USA) was used to confirm total RNA quantity and integrity. Purified samples were then prepared using a standard polyA-enriched library preparation protocol (NEBNext Ultra II Directional RNA-Seq Kit standard protocols) implemented by ebiogen (Seoul, Korea). Ribosomal RNA was removed prior to proceeding. Sequencing was performed using the Illumina NovaSeq 6000 that generates paired end reads of 101 bp. Illumina Casava1.8 software used for basecalling. Sequenced reads were trimmed for adaptor sequence, and masked for low-complexity or low-quality sequence using fastx trimmer, then mapped to UCSC mm10 whole genome (*Mus musculus* genome) using Tophat. The mapped reads (FPKM) were further analyzed with Cufflink to calculate the level of gene expression. The sequencing data were deposited into GEO repository with the accession number GSE168379. For detailed differentially expressed gene (DEG) analysis, raw gene counts of the transcriptome were normalized by size factors and compared between wild-type and Nsrp70-KO samples by negative binomial tests using the R DESeq2 package. Based on significantly differentially expressed genes (adjusted *P*-value < 0.05), we performed gene ontology enrichment tests among up-regulated or down-regulated genes using the modified Fisher's Exact tests with the RDAVIDWebService package (*P*-value < 0.01). The alternative splicing events were analyzed using the MISO package with the annotation of all known alternative splicing events (20). The significant differentially spliced events were determined by Bayes’ factor (BF) and Percent spliced in values (PSI, Ψ) (BF ≥ 10 and PSI ≥ 0.2). Gene ontology and KEGG analysis were performed using the functional annotation tool of DAVID (21) to search for enriched pathways of associated alternative splicing events. The functional associations of NSrp70 targets were analyzed using protein interaction dada from the STRING database (22), generating a set of functional interaction networks.

### Reverse transcription PCR (RT-PCR) and real-time quantitative RT-PCR (qRT-PCR)

Total RNA was isolated from cells or homogenized tissues of C57BL/6 mice with TRIzol reagents (Molecular Research Center, Cincinnati, OH, USA) and reverse transcribed using RT-Premix (Intron Biotechnology). PCR was performed with the respective forward and reverse sequence pairs as indicated in Supplementary Table S1. The expression levels of mouse *Cdk1, Cdca3, Rpl26, Uba52, Atp5pd*, Ndufs5*, Fyn, Plcg1, Ccr7, Junb, Ptk2b* and *Bag6* were evaluated by qRT-PCR. Amplification was performed in a StepOne real-time PCR system (Applied Biosystems, Norwalk, CT, USA) for continuous fluorescence detection in a total volume of 10 μl of cDNA/control and gene-specific primers using SYBR Premix Ex Taq (TaKaRa Bio). The mRNA levels of the target genes were normalized relative to those of *Gapdh* using the following formula: relative mRNA expression = 2^−^(ΔCt of target gene − ΔCt of *^Gapdh^*), where Ct is the threshold cycle value. In each sample, the expression of the analyzed gene was normalized to that of *Gapdh* and described as the mRNA level relative to *Gapdh*.

### RNA immunoprecipitation

RNA immunoprecipitation (RIP) experiments were performed using a Magna RIP RNA-Binding Protein Immunoprecipitation Kit according to the manufacturer's instructions (Millipore) using a modified version of a previously described method (23). Briefly, HEK293T cells (1 × 10^7^) transfected with the indicated constructs were lysed on ice in complete RIP lysis buffer (Millipore). GFP or IgG control antibodies were pre-coated onto magnetic beads protein A/G for 30 min at room temperature. Antibody-coated beads were washed two times with RIP wash buffer (Millipore) using a magnetic separator. Protein lysates were then incubated with rabbit anti-GFP- or control IgG-coated beads overnight at 4°C. The immunoprecipitated samples were then centrifuged and washed with ice-cold RIP wash buffer six times using a magnetic separator. After the final wash, the RNA–protein complexes were dissociated from the beads by incubating in proteinase K buffer (Millipore) for 30 min at 55°C. The supernatants were collected using a magnetic separator. RNA extraction from the supernatant was performed with phenol:chloroform:isoamyl alcohol (125:24:1) (Sigma-Aldrich) and precipitated using ethanol supplemented with salt solution I, salt solution II, and precipitation enhancer overnight. The RNA pellets were then washed and suspended with RNAse-free water. The cDNA was transcribed from total RNA using Reverse Transcript PCR Premix (Intron Biotechnology). PCR was performed as described above.

### *In vivo* splicing assays

*In vivo* splicing assays were performed as previously described (24). Briefly, a splicing reporter minigene was co-transfected with an increasing amount of wild-type GFP_NSrp70 construct, together with mCherry-tagged SRSF1 in HEK293T cells. Empty plasmids were added to ensure that the same amount of DNA was transfected. Forty-eight hours after transfection, total RNA was extracted from cells using the Total RNA isolation reagent (Molecular Research Center). The cDNA was transcribed from 2 μg of total RNA using Reverse transcript PCR premix (Intron Biotechnology). PCR for the FAS minigene was performed as previously described (19). The PCR products were analyzed with a 1–1.5% agarose gel and the splicing pattern was quantified using ImageJ (ImageJ is a public domain java image processing program inspired by National Institutes of Health Image).

### Flow cytometry analysis

Thymus, spleen and lymph nodes were dissected and passed through a 40 μm cell strainer (BD Biosciences). The passed cells were washed with PBS and remaining erythrocytes were lysed with a commercially available hemolysis buffer (Morphisto, Frankfurt am Main, Germany). Isolated cells were washed and stained for flow cytometry with antibodies against CD4, CD8, CD25, CD24, Foxp-3, TCR-β, CD69, TCR-ϵ and TCR-ζ for 30 min at 4°C. Data were acquired on a FACSCanto (BD Biosciences, San Jose, CA, USA) and analyzed with FlowJo software (Tree Star, Ashland, OR, USA). Appropriate isotype controls were included and gates were set according to isotype-matched controls. For intracellular staining, cells were fixed and permeabilized with intracellular fixation and permeabilization buffer. Fixed cells were stained for flow cytometry with antibodies against Bcl-2 and Ki67 for 30 min at 4°C. Total cellularity was determined by counting the live cells. Absolute cell numbers were calculated on the basis of the percentage of each population and represented as the mean ± SD. The mice in these experiments were 6∼8 weeks of age.

### Isolation of oocyte, morula, and embryos and immunofluorescence staining

C57BL/6 mice were injected with 10 IU/mouse of PMSG (pregnant mare serum gonadotrophin). After 48 h, mice were re-stimulated with 10 IU/mouse of hCG (human chorionic gonadotropin). After 13 h, mice were terminated to obtain oocytes. Mouse embryonic day is related to the female presence of a vaginal plug indicating that mating occurred. Day 1 of pregnancy was defined as the day on which the vaginal plug was observed. At 2 d post coitum (dpc), mice were terminated to obtain morula; in the same way we could obtain E11.5 (11.5 dpc) to E18.5 (18.5 dpc) stages of embryos.

### Freeze sectioning and confocal microscopy

Embryos were snap-frozen in isopentane precooled close to its freezing point in liquid nitrogen and then 5-μm thick sections were prepared and mounted onto poly-l-lysine coated glass slides. Sections were air-dried under a fan for 2–24 h, after which the embryos were incubated first with anti-NSrp70 antibody and then with the FITC-conjugated secondary antibody for NSrp70 and TRITC-phalloidin for actin, for 30 min at 37°C. For frozen sections of immune related tissues, mice were anaesthetized, and the thymus, spleen and lymph node of each mouse was collected and fixed with 4% paraformaldehyde for 4 h, followed by treatment with a 30% sucrose solution in PBS overnight at 4°C. Tissue samples were embedded in optimal cutting temperature (OCT) medium and stored at –80°C. The samples were sectioned to a 7-μm thickness. The slides were examined using a FV1000 confocal laser scanning microscope (Olympus, Tokyo, Japan) equipped with 40×, 60× and 100× objectives.

### PCR genotyping assay of progenies

Tails of progenies were lysed in 1.5 ml tubes containing tail lysis buffer (DirectPCR Lysis Reagent, VIAGEN Biotech), and 2 μg lysate was used for PCR genotyping. The PCR primers for the wild-type allele were located in exon 1 (21T 93–1: 5′- ATATACACGTCGGCGTCAGC-3′) and exon 2 (21T93-3R: 5′-CAAAATAAGCC CATACCTGCGTAA-3′), and the PCR primers for the trap allele were located between exon 1 and exon 2 (21T93-9: 5′-GAAGAGAGGCCCATTGGTTG-3′, SA-5SA: 5′-GGGCAAGAACATAAAGTGACC-3′). Primers 21T93-1 and 21T93-3R were used to amplify 1,737 base pairs of intron 1 (35 cycles of 1 min at 94°C, 1 min at 58°C and 2 min at 72°C), and primers 21T93-9 and SA-5AS were used to amplify 597 bp that consisted of the partial trap vector and the intron 1 sequence (35 cycles of 1 min at 94°C, 15 s at 56°C, and 30 s at 72°C).

### Ca^2+^ flux analysis

Thymocytes and lymphoblasts were first labeled for 1 h at 37°C with 4 μg/ml Fluo-3/AM (Invitrogen) and then washed and resuspended with ice-cold PBS. Cells (3 × 10^6^) were labeled on the surface for 30 min on ice with PE-conjugated anti-CD4 (H129.19; BD Biosciences) and PE-Cy3-conjugated anti-CD8 (53–6.7; BD Biosciences), and then incubated with biotinylated anti-CD3 (5 μg/ml; 145–2C11) and anti-CD28 (5 μg/ml; 37.51). Labeled cells were warmed for 20 min at room temperature and then crosslinked with streptavidin (25 μg/ml) or stimulated with PMA (phorbol 12-myristate 13-acetate) and ionomycin immediately before flow cytometry. Mean fluorescence ratios were plotted after analysis with FlowJo software (TreeStar, Ashland, OR, USA).

### *In vitro* development assay and thymocyte stimulation

The *in vitro* development assay was performed essentially as described (25). Sorted CD69^–^ DP thymocytes were resuspended in RPMI 1640 and incubated for 20 h in wells non-coated or coated with anti-TCRβ (MABF931) and anti-CD2 (130–100-617). Cells were extensively washed and either analyzed immediately by flow cytometry (stimulatory culture) or incubated for 20 h in the same medium prior to analysis by flow cytometry (recovery culture). For treatment of cell cycle inhibitor and HSF1 inhibitors, sorted CD69^+^ DP thymocytes were resuspended in RPMI 1640 containing the indicated inhibitor and processed using the *in vitro* development assay, as described above. To measure intracellular signaling, DP thymocytes were stimulated with plate-bound anti-CD3 (5 μg/ml) and anti-CD28 (5 μg/ml) for 0, 5 and 20 min.

### Colony formation assay

Colony formation assays were performed as previously described (26). The EO771 cells expressing NSrp70, SRSF1 or control vector (300–500 cells per dish) were seeded in a six-well plate and incubated at 37°C in a humidified incubator for 2 weeks. Colonies were fixed with 4% paraformaldehyde and stained with 0.01% crystal violet and the number of colonies was counted using ImageJ.

### Tumor animal models

Control B16F10 melanoma cells (2 × 10^6^ cells/0.1 ml media per mouse) were injected subcutaneously into the two dorsal flank regions of *Nsrp1****^f/f^***(WT) and *Nsrp1*^f/f^CD4Cre (KO) mice (6–8 weeks old). After 2 weeks, mice were sacrificed, tumors were dissected, photographed, and weighed.

### Computational resources

*TCGA database and cBioPortal*. cBioPortal for Cancer Genomics (http://cbioportal.org) provides an open-access web resource for exploring, visualizing and analyzing multidimensional cancer genomic data from TCGA (27). In the present study, TCGA PanCancer Atlas studies provided by default on the cBioPortal were selected for further analysis of NSRP1 gene alterations. The cancer types summary and survival tabs were applied according to the online instructions of cBioPortal.

### Statistical analyses

Mean values were calculated using data taken from at least three independent experiments conducted on different days. Where significance testing was performed, unpaired Student's *t* tests and one-way analysis of variance tests were used. Differences between groups were considered significant at *P*-value <0.05.

## RESULTS

### NSrp70 is expressed in early developmental cells as well as in lymphoid cells

**Figure 1.**
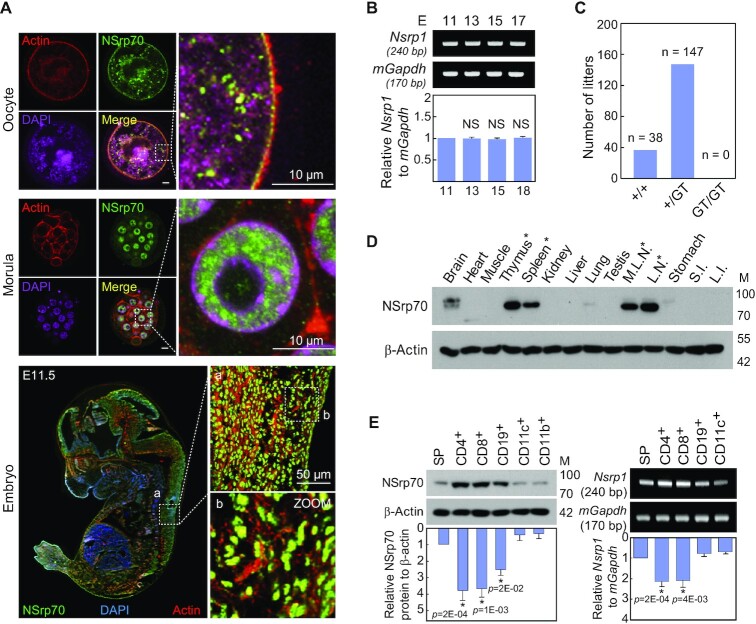
NSrp70 is preferentially expressed in early embryonic tissues and in lymphoid cells. (**A**) Immunohistochemistry for NSrp70. Oocyte (top), morula (middle), and fetal embryo E11.5 (bottom) were isolated from WT mice, stained with anti-NSrp70 antibody followed by FITC-conjugated 2^nd^ antibody (green), TRITC-phalloidin (red), and DAPI (magenta and blue), and then visualized by confocal microscopy. (**B**) Fetal embryos from gestation day 11.5, 13.5, 15.5 and 17.5 mice were isolated. *Nsrp1* mRNA was detected by RT-PCR (blots) and density was represented by bar graph. m*Gapdh* was used as a loading control. E, embryonic day; bp, base-pair. (**C**) Intercrossing the heterozygous *Nsrp1*^+/GT^ mouse produced no offspring homozygous for the allele containing the gene-trap vector. GT, gene-trap vector; +/+, wild-type; +/GT, heterozygous *Nsrp1*^+/GT^; GT/GT, homozygous *Nsrp1*^GT/GT^. (**D**) Tissue distribution of NSrp70 was determined by western blot analysis in 8-week old mice. M.L.N, mesenchymal lymph node; L.N., lymph node; S.I., small intestine; L.I., large intestine; M, molecular mass (KDa). (**E**) Western blot (left) and RT-PCR (right) for NSrp70 in mouse immune cells. β-actin and m*Gapdh* were shown as loading controls. SP, splenocytes. All data shown are representative of three independent experiments. The bar graphs indicate the mean ± standard deviation of the indicated protein blot or RNA gel densitometry presented with respect to β-actin or *mGapdh* (B and E).

In a previous report, a knockout of *Nsrp1* (*Nsrp1^GT/GT^*), a designated gene name for NSrp70, generated by the gene-trap method resulted in embryonic lethality, suggesting an important role of NSrp70 in early embryogenesis (3). Consistent with this, NSrp70 was detected in oocytes and at the morula stage and entire areas in the developmental embryo at day 11.5 (Figure 1A). *Nsrp1* was constitutively expressed in all stages of embryo development (Figure 1B). Off-spring lethality indicates that NSrp70 plays a major role in development, while the detailed mechanism is completely unknown (Figure 1C).

However, NSrp70 was highly and selectively expressed in primary and secondary lymphoid organs, such as thymus, spleen, mesenteric lymph nodes, and lymph nodes (Figure 1D, *asterisk*), demonstrating that this protein may play a role in immunity after birth. Interestingly, NSrp70 and its gene *Nsrp1* were predominantly expressed in lymphocytes such as CD4^+^ and CD8^+^ T cells, and CD19^+^ B cells (Figure 1E), suggesting that NSrp70 may be a potential lineage-specific regulator of lymphocyte development.

### NSrp70 deletion results in severe thymic defects

**Figure 2.**
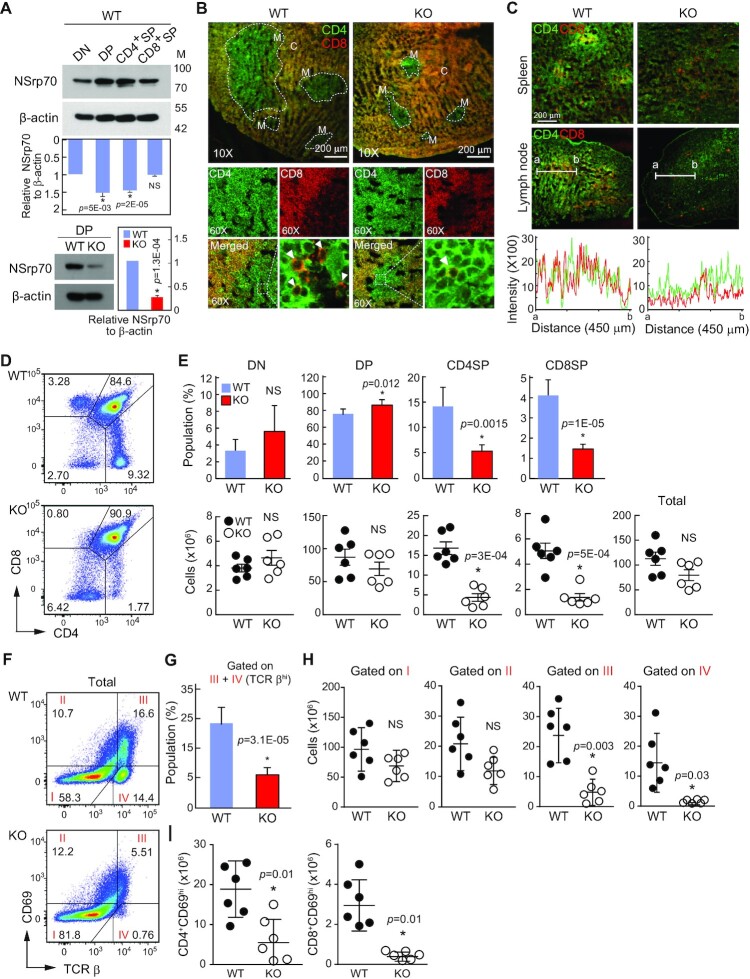
Development of CD4^+^ and CD8^+^ SP thymocytes is impaired in *Nsrp1*^f/f^CD4Cre mice. (**A**) Western blot of NSrp70 expression in thymocyte subsets from *Nsrp1*^f/f^ (WT) and *Nsrp1*^f/f^CD4Cre (cKO) mice. DN, double negative; DP, double positive; SP, single positive; WT, *Nsrp1*^f/f^ mouse; KO, *Nsrp1*^f/f^CD4Cre mouse. The bar graphs indicate the mean ± standard deviation (SD) of the indicated protein blot densitometry presented with respect to β-actin. (B and C) Staining for CD4 (green) and CD8 (red) in thymus (**B**) and in spleen and lymph node (**C**) from 6-week-old WT and KO mice. White arrowheads: CD8^+^ SP thymocytes in medulla. M, medulla; C, cortex. Original magnification, ×10. The fluorescent intensity of CD4^+^ and CD8^+^ thymocytes was quantified (C, bottom). (**D**) Flow cytometric analysis of CD4 and CD8 on thymocytes. (**E**) Quantification of average percent (top) and cell numbers (bottom) of DN, DP, CD4^+^ or CD8^+^ SP thymocyte subpopulations. Each black and white circle in graphs of cell numbers represents an individual mouse. The bar graphs (top) and small horizontal lines (bottom) indicate the mean ± SD. *, meaningful *P*-value; NS, non-significant *P*-value. (**F**) Surface staining of TCRβ and CD69 on total thymocytes. (**G**–**I**) Quantification of TCRβ^hi^ thymocytes population gated on III and IV (G), total cell numbers gated on I–IV gate plots (H), and CD4^+^CD69^hi^ and CD8^+^CD69^hi^ cells (I) from panel (F). The bar graphs indicate the mean ± SD of populations. hi, high expression. Each black and white circle in graphs represents an individual mouse. The small horizontal lines indicate the mean ± SD. All data shown are representative of three independent experiments.

We generated mice carrying a germ line-transmitted *Nsrp1*-floxed allele using Cre-*loxP* and FLP-*frt* gene recombination systems. *Nsrp1*^f/f^ mice cross-mated with CD4Cre transgenic mice could generate *Nsrp1*^f/f^CD4Cre mice (Supplementary Figure S1A). *Nsrp1*^f/f^CD4Cre (NSrp70-conditional-knockout, cKO) mice were viable and showed no overt signs of weight difference, nor differences in the sizes of immune organs such as thymus, spleen, and lymph node as compared to those of the wild-type (WT, *Nsrp1*^f/f^) (Supplementary Figure S1B and C). NSrp70 was upregulated at the DP stage, but significant reduction of NSrp70 was confirmed in NSrp70-cKO thymocytes (Figure 2A). Although there was no sign of morphological changes, interestingly, smaller areas of the medulla accompanied by significant reduction of CD4^+^ and CD8^+^ T cell zones were shown in NSrp70-cKO thymus (Figure 2B, *arrowheads*, and Supplementary Figure S1C). In addition, CD4^+^ and CD8^+^ SP signals were low in the spleen and lymph nodes (Figure 2C), suggesting that thymocytes are not properly developed at the DP stage.

We assessed the populations of thymocytes to determine the populations and numbers of CD4^+^ and CD8^+^ cells. In agreement with the results of the thymic tissue section, a significant reduction of CD4 and CD8 SP thymocytes in NSrp70-cKO mice was observed as compared with that of WT mice, while total numbers of thymocytes were not altered substantially (Figure 2D and E). In addition, the populations and numbers of DN and DP thymocytes were not dramatically altered (Figure 2D and E).

We observed a considerable decrease in the number of CD4^+^ and CD8^+^ SP thymocytes in NSrp70-cKO mice (Figure 2E). To clarify at which stage thymocyte development was blocked, we quantified cells at four distinct developmental stages based on the expression of the maturation marker TCRβ and the positive selection marker CD69. Although the population and numbers of TCR-β^lo^CD69^lo^ cells (Gate I: mostly DN and preselected DP cells) and TCRβ^int^CD69^int^ cells (Gate II: thymocytes undergoing selection) were unchanged, the population and numbers of TCRβ^hi^CD69^hi^ cells (Gate III: post-positive selection thymocytes) and TCRβ^hi^CD69^lo^ cells (Gate IV: mature SP cells ready for export to the periphery) were significantly reduced in NSrp70*-*cKO mice (Figure 2F – H). Accordingly, the numbers of CD4^+^CD69^hi^ and CD8^+^CD69^hi^ thymocytes were reduced in NSrp70*-*cKO mice as compared to those in control mice (Figure 2I). As a result, mature (CD24^lo^) TCRβ^hi^CD4^+^ or TCRβ^hi^CD8^+^ SP thymocytes were significantly reduced (Supplementary Figure S2A and B). Taken together, these results indicate that NSrp70 deletion results in severe defects in the late development of DP thymocytes during positive selection. However, despite such a marked reduction in SP thymocytes, the reason for the small change in the size (Supplementary Figure S1C) and cellularity (Figure 2E) of the thymus is probably due to the fact that CD4 and CD8 SP thymocytes represent only approximately 10% of the total thymocytes in the thymus.

### NSrp70 regulates alternative splicing (AS) events of pre-mRNA splicing factors in DP thymocytes

**Figure 3.**
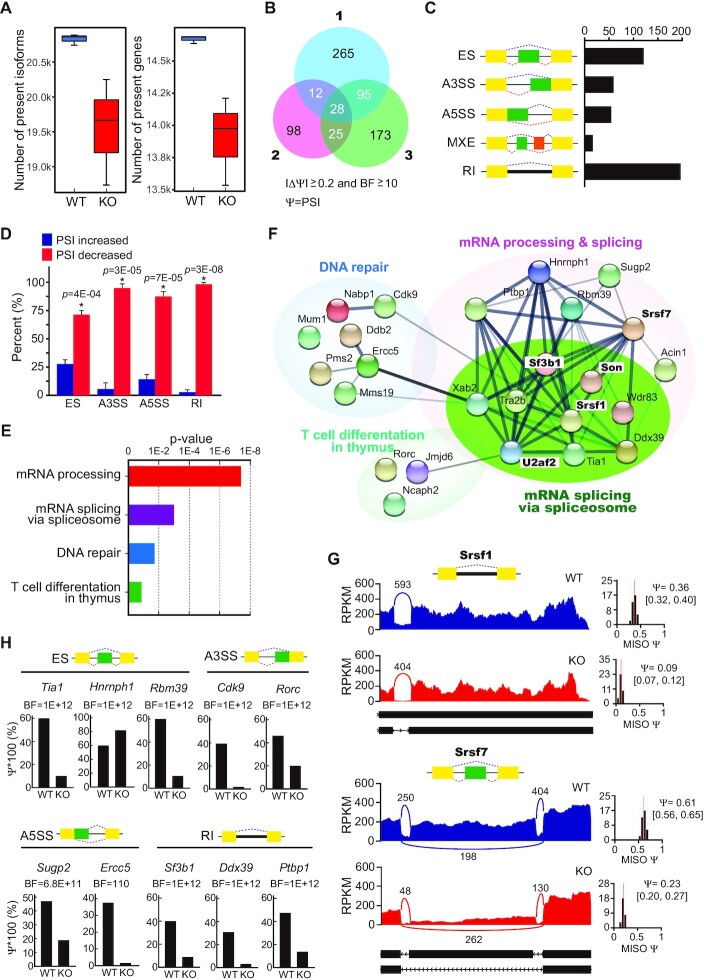
Global alternative RNA splicing changes in NSrp70-cKO DP thymocytes. (**A**) The number of isoforms and genes present in the given samples. We checked the number of isoforms and genes present in given wild-type and KO samples and found significant reductions of the numbers of isoforms and genes in DP thymocytes (6.13% and 5.5% reductions, respectively; Wilcoxon rank sum tests, one-sided, *P*-values ≤ 0.05). (**B**) Venn diagram of the number of significantly alternative spliced-genes from three mRNA-seq datasets of *Nsrp1*^f/f^ (WT) and *Nsrp1*^f/f^CD4Cre (KO) DP thymocytes. PSI, Percent spliced in; BF, Bayes’ factor. (**C**) Quantification of the different alternative splicing events affected by NSrp70. (**D**) The relative percent of each alternative splicing event affected by NSrp70. The small horizontal lines indicate the mean ± standard deviation *, meaningful *P*-value. (**E**, **F**) Gene ontology analysis (E) and functional association network (STRING) (F) of alternative spliced-targets affected by NSrp70-cKO. Fisher exact *P*-values were plotted for each enriched functional category. (**G**) Sashimi plot of alternative exons affected by NSrp70. Genes were chosen to represent both an increase and a decrease of PSI, and the numbers of exon junction reads are indicated. (**H**) PSI and BF values of different types of NSrp70-regulated alternative splicing events based on MISO analysis.

Previously, we reported that NSrp70 modulates the alternate splice site selection of several splicing reporter mini-genes (3). To gain further insights into NSrp70-regulated AS events related with its physiological functions, we performed high-throughput sequencing of mRNA (mRNA-seq) with deep sequencing coverage (∼60 million, 100-nt paired-end reads) after sorting of DP thymocytes from WT and NSrp70-cKO mice. Strikingly, we observed that the number of isoforms and genes were markedly reduced (6.13% and 5.5% reductions, respectively; Wilcoxon rank-sum tests, one-sided, *P*-values ≤ 0.05) in DP thymocytes from NSrp70-cKO mice (Figure 3A), implying remarkable changes in the alternative splicing landscape. For a better characterization of the changes in the AS events, we analyzed AS changes of individual transcripts using mixture-of-isoforms (MISO) models (20) and identified each 400, 163 and 321 NSrp70-regulated AS events with an obvious change in PSI values (PSI ≥ 0.2) (Figure 3B). Of these, 160 genes that commonly appeared in two or more sets were selected (Figure 3B). The alternatively spliced genes are listed in Supplementary Table S2. We observed 473 NSrp70-regulated AS events, including exon skipping (ES), alternative 3′/5′ splice sites (A3SS/A5SS), mutually exclusive exons (MXE), and retained intron (RI) (Figure 3C). Subsequent analysis revealed that most AS events were negatively regulated in NSrp70-cKO DP thymocytes (decreased PSI value by NSrp70 KO) (Figure 3D).

When analyzing the cellular functions of NSrp70-regulated AS events using gene ontology (GO), we found that NSrp70 affects genes in the mRNA processing and splicing, DNA repair, and T cell differentiation pathways (Figure 3E). These results are direct evidence that NSrp70 is an RNA binding factor known to regulate alternative splicing in the spliceosome of nuclear speckles (3,19). NSrp70-regulated splicing targets were heavily connected with mRNA processing and splicing interaction networks, as judged by the Search Tool for the Retrieval of Interacting Genes/Proteins (STRING) (Figure 3F). Intriguingly, NSrp70 targets are enriched with well-known cell cycle-related splicing factors, such as *Srsf1, Srsf7* and *Son* (16,17,28), and spliceosome-related factors, such as *Sf3b1* and *U2af2* which are important for splice site recognition (18). The read tracks of *Srsf1* and *Srsf7* and other splicing target genes are shown as examples (Figure 3G and H).

### NSrp70 sequesters pre-mRNA splicing factors, including SRSF1, into the nuclear speckles and counteracts SRSF1

**Figure 4.**
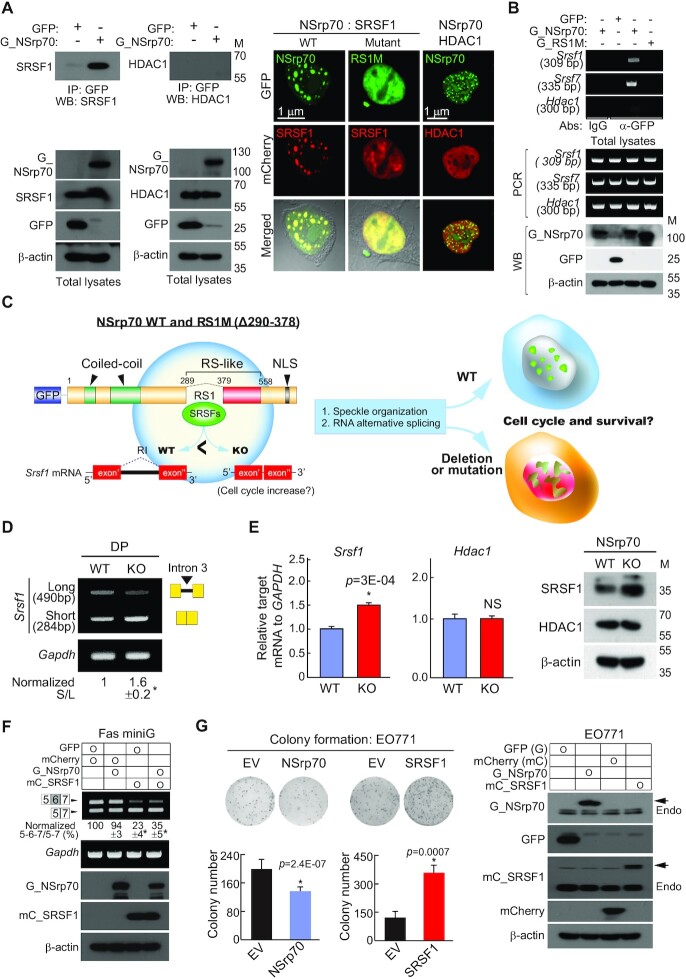
NSrp70 controls the expression of SRSF1 and counteracts mutually. (**A**) Immunoprecipitation (left) and immunofluorescence images (right) of NSrp70 and SRSF1. HEK293T cells were transfected with GFP (empty vector), GFP_NSrp70 (G_NSrp70), or RS1M mutant. In some cases, mCherry-fused SRSF1 and HDAC1 were co-transfected. Samples were immunoprecipitated and blotted with antibodies against the indicated proteins (left). IP, immunoprecipitation; WB, western blotting; M, molecular mass (kDa). Fluorescence signals were visualized under a confocal microscope (right). Magnification, 100×. Results are representative of three independent experiments. (**B**) RIP assay for validation of NSrp70-binding mRNA. The samples in (A) were immunoprecipitated with the indicated antibodies (anti-rabbit IgG or anti-GFP). RNAs purified from the IP samples and total lysates were determined by RT-PCR. bp, base pair. (**C**) Schematic diagram of wild-type NSrp70 and RS1M mutant. Potential regulation of alternative splicing and speckle organization by NSrp70 and its effect on T cell development. NLS, nuclear localization sequence. (**D**) RT-PCR analysis of different SRSF1 isoforms in *Nsrp1*^f/f^ (WT) and *Nsrp1*^f/f^CD4Cre (KO) DP thymocyte. (**E**) Real-time quantitative PCR (left) and western blot analysis (right) for SRSF1 and HDAC1 from WT and KO DP thymocytes. *Gapdh* and β-actin were shown as the loading controls. *, meaningful *P*-value. (**F**) NSrp70 counteracts SRSF1 *in vivo* splicing assay. HEK293T cells were co-transfected with the indicated constructs and Fas minigene. Exon inclusion or exclusion was determined by RT-PCR (top). The ratio of exclusion or inclusion of Fas exon 6 is shown as the normalized ratio (%) (middle). mCherry, empty vector; mC_SRSF1, mCherry_SRSF1. (**G**) NSrp70 effects on the proliferation of EO771 cancer cells. The cells were stably transfected with NSrp70, SRSF1, or a control vector and analyzed by colony formation assays with mean ± standard deviation of relative colony numbers plotted (left). Expression of NSrp70 and SRSF1 were confirmed (right). All data shown are representative of three independent experiments. *, meaningful *P*-value.

Interestingly, we previously demonstrated that NSrp70 physically interacts with SRSF1 and SRSF2 (3). In the present study, we further corroborated the binding of NSrp70 to SRSF1, but not to histone deacetylase 1 (HDAC1) (29), a transcriptional regulator known to be involved in T cell development (Figure 4A). In addition, NSrp70 directly binds to the mRNA of *Srsf1* and *Srsf7*, as determined by an RNA immunoprecipitation (RIP) assay (Figure 4B). In a previous report, we identified that the protein-protein interaction site of NSrp70 is the RS1 region, as shown in the schematic cartoon in Figure 4C (3,19). Deletion of the RS1 region (RS1M) caused complete loss of NSrp70 localization in the nuclear speckles and showed a disturbed pattern in the nucleus (3,19). Interestingly, co-transfection of RS1M caused failure of speckle localization of SRSF1 (Figure 4A). Disturbed nuclear speckle localization is also observed with other NSrp70-binding proteins, such as SON, U2AF1, and hnRNP U, but not with U170K, a nuclear speckle protein that does not bind NSrp70 (Supplementary Figure S3). Since spatiotemporal subnuclear organization of the pre-mRNA processing machinery is essential for the proper maturation of protein-coding mRNAs (1), these results suggest that NSrp70 is associated with the expression and function of other pre-mRNA splicing factors, they leading to altered expression of many downstream genes involved in T cell development (Figure 4C).

Among various NSrp70-regulated target splicing factors (Figure 3E and F), SRSF1 is particularly interesting, because it is a well characterized oncogenic factor that promotes tumorigenesis through multiple pathways (29). In addition, interestingly, retention of intron (RI) is most often associated with down-regulation of gene expression via non-sense-mediated decay (NMD) (30,31). Since the RI form of *Srsf1* mRNA was significantly reduced in NSrp70-cKO DP thymocytes in the present study (Figures 3G and 4D), we expected that *Srsf1* mRNA and its canonical protein isoform would be increased in NSrp70-cKO DP thymocytes. As expected, the increased *Srsf1* mRNA and protein were seen in NSrp70-cKO DP thymocytes (Figure 4E).

Since SRSF1 is an oncogenic factor, we further investigated whether NSrp70 has an opposite role to SRSF1 in cells. Overexpression of NSrp70 suppressed SRSF1-mediated alternative splicing of the Fas minigene (Figure 4F). In addition, colony formation assay revealed that overexpression of NSrp70 significantly reduced colony formation in E0771 breast cancer cells, whereas SRSF1 increased it (Figure 4G).

### Aberrant gene expression related to the cell cycle, translation, and subcellular locations associated with alternative splicing in NSrp70-cKO DP thymocytes

**Figure 5.**
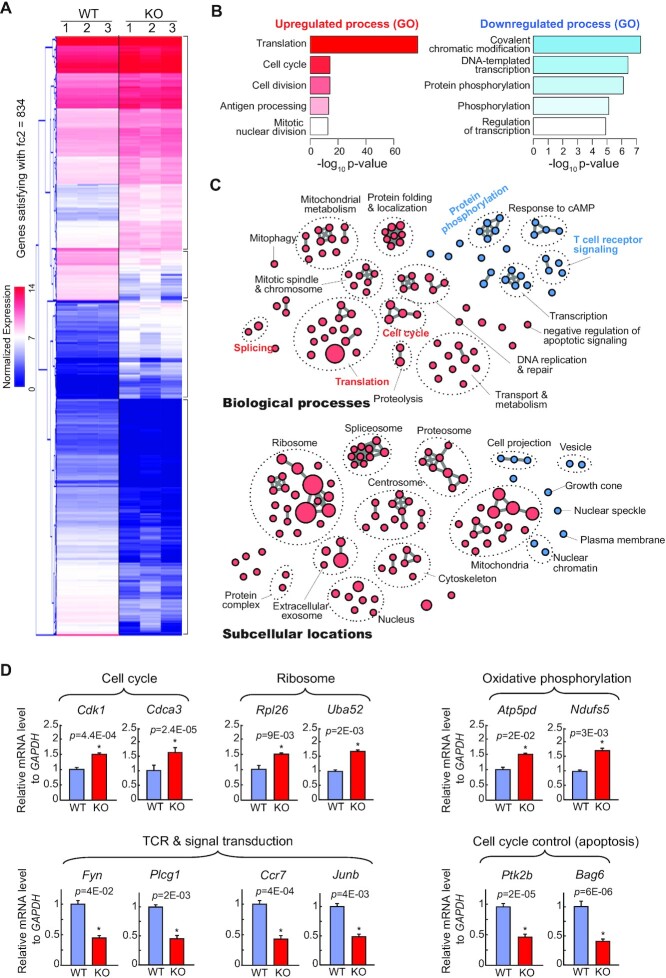
Aberrant gene expression related to the cell cycle and TCR intracellular signal transduction in NSrp70-cKO DP thymocytes. (**A**) Hierarchical clustering of gene expressions with more than two-fold changes. We found distinct gene clusters of up- or down-regulation in Nsrp70-cKO DP thymocytes. The heat-map for gene expression patterns was generated using the Multi Experiment Viewer (MeV) software. Genes with red and blue colors indicate higher and lower expressions, respectively. fc2, 2-fold change. (**B**) Top-5 most significantly up-regulated or down-regulated biological process terms from significantly differentially expressed genes (negative binomial tests) based on gene ontology enrichment tests (modified Fisher's exact tests from DAVID). (**C**) The global map of significantly up-regulated (pink) or down-regulated (skyblue) gene ontology terms (biological processes and subcellular locations, top and bottom, respectively) by gene ontology enrichment tests (*P*-value < 0.01). Like in the Enrichment Map (54), we clustered enriched biological processes and subcellular locations as connected in the map if they shared genes in significant numbers (Jaccard > 0.25). (**D**) Real-time quantitative PCR analysis for the validation of NSrp70-regulated targets. m*Gapdh* was used as the loading control. *, meaningful *P*-value. All data shown are representative of three independent experiments.

We subsequently analyzed how NSrp70-cKO affects global gene expression. Based on hierarchical clustering of gene expressions, we found distinct clusters of up- or down-regulated expressions in the Nsrp70-cKO group (Figure 5A; genes with more than 2-fold changes were shown). Based on the R DESeq2 package, we identified 834 significantly up-regulated genes and 295 down-regulated genes (adjusted *P*-value < 0.05) (Supplementary Table S3) (32).

Interestingly, based on GO enrichment tests of significant up- or down-regulated genes (Figure 5B and Supplementary Table S4), we found that biological processes related to cell proliferations (e.g. cell cycle) and the synthesis of cellular building blocks and energy (e.g. translation, DNA replication and repair, and mitochondrial metabolism) were significantly up-regulated, whereas transcription, protein phosphorylation, T cell receptor signaling pathway, and the negative regulation of apoptotic signaling were significantly down-regulated (Figure 5C and Supplementary Table S4; *P*-value < 0.01). Furthermore, we also identified subcellular locations significantly enriched among up- or down-regulated genes (Figure 5C and Supplementary Table S5; *P*-value < 0.01). For example, we found that subcellular locations for macromolecular synthesis and cell cycle processes (e.g. ribosome, cytoskeleton, and centrosome) were significantly up-regulated, whereas major subcellular locations associated alternative splicing, such as nuclear chromatin and nuclear speckle were down-regulated.

Quantitative RT-PCR confirmed the results of RNA-seq analysis and validated the increased expression of *Cdk1, Cdca3, Rpl26, Uba52, Atp5pd* and *Ndufs5*, which are related to the cell cycle, translation, and metabolism, and the decreased expression of *Fyn, Plcg1, Ccr7, Junb, Ptk2b* and *Bag6*, which are related to TCR signal transduction and anti-apoptosis (Figure 5D). Taken together, these results strongly suggest that NSrp70 function is closely linked to cell cycle progression and building block synthesis during the transition of DP to SP thymocytes.

### Deletion of NSrp70 induces uncontrolled cell proliferation followed by apoptotic cell death

**Figure 6.**
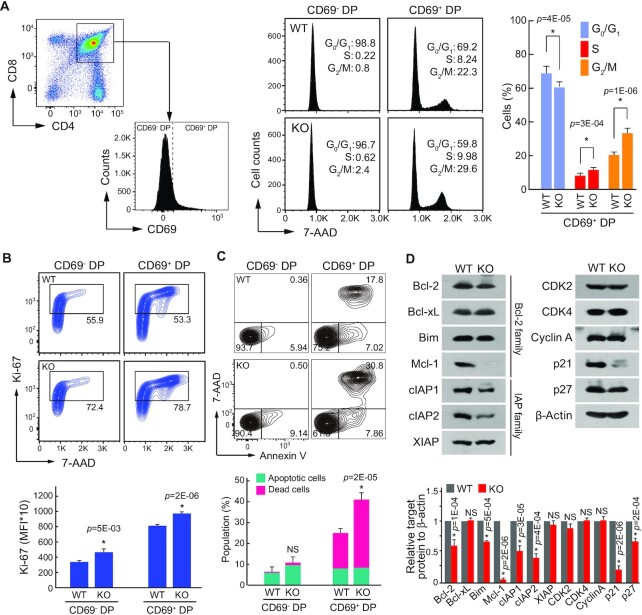
Deletion of NSrp70 induces uncontrolled cell proliferation followed by apoptotic cell death. (**A**) Cell cycle analysis of DP thymocytes from *Nsrp1*^f/f^ (WT) and *Nsrp1*^f/f^CD4Cre (KO) mice. Populations of CD69^–^ or CD69^+^ thymocytes were gated from CD4 and CD8 DP thymocytes (left) and were analyzed for DNA content by 7-ADD intensity (right). The bar graphs indicate average ± SD of different cell cycle stages population. *, meaningful *P*-value. (**B**, **C**) Analysis of cell proliferation by Ki-67 staining and apoptotic cell death by annexin V and 7-ADD. DP thymocytes from (A) were stained with anti-Ki-67 antibody and 7-ADD (B) or annexin V and 7-ADD (C) and analyzed by flow cytometry. Bar graphs indicate mean fluorescence intensities (MFI) (B, *bottom*). Annexin V^+^ populations represent early apoptotic cells and annexin V^+^ and 7-ADD^+^ populations represent dead cells (C). The bar graphs indicate average ± SD of apoptotic and dead thymocyte populations. NS, non-significant *P*-value. (**D**) Western blot of cell cycle and cell death-related proteins in samples extracted from WT and KO thymocytes. β-actin served as a loading control. The bar graphs indicate average ± SD of indicated protein blot densitometry presented relative to β-actin. All data shown are representative of three independent experiments.

Next, we investigated whether the increased expression of cell cycle regulators in NSrp70-cKO DP thymocytes is related to increased cell cycle activity *in vivo*. To this end, total DP thymocytes were gated by CD69^–^ (before positive selection) and CD69^+^DP (post-positive selection), and then the cells were assessed for cell cycle activity and apoptotic cell death. Most CD69^–^ DP thymocytes showed G_0_/G_1_ arrest of the cell cycle (Figure 6A). In contrast, NSrp70-cKO CD69^+^ DP thymocytes showed increased populations of S and G_2_/M phases as compared to WT (Figure 6A). Consistently, NSrp70-deficient CD69^+^ DP thymocytes contained higher numbers of Ki-67-positive cells than that of WT (Figure 6B), demonstrating an active cell proliferation at the CD69^+^ DP stage. Higher numbers of Ki-67-positive cells were also seen in NSrp70-cKO CD69^–^ DP thymocytes (Figure 6B), demonstrating that loss of NSrp70 in thymocytes triggers uncontrolled cell proliferation. Interestingly, large populations of NSrp70-cKO CD69^+^ DP thymocytes underwent apoptotic cell death (Figure 6C). This event was accompanied by reduced expression of p21 (Figure 6D), which inhibits cell cycle progression and apoptosis (33), and Bcl-2 and Mcl-1 (Figure 6D), which play a role in survival and apoptosis (34).

Knockdown of NSrp70 also showed upregulation of candidate cell cycle regulators in non-immune HEK293T cells (Supplementary Figure S4A), suggesting that the role of NSrp70 generally contributes to the expression of cell cycle regulators in cells. In contrast, NSrp70-cKO did not affect the expression of genes known to be associated with specific stages of T cell development, as corroborated by RT-PCR (Supplementary Figure S4B and C). Collectively, these results demonstrate that NSrp70 is a critical regulator of cell cycle arrest and survival during TCR-mediated positive selection.

### Deletion of NSrp70 results in defective survival signals following TCR activation in CD69^+^ DP thymocytes

**Figure 7.**
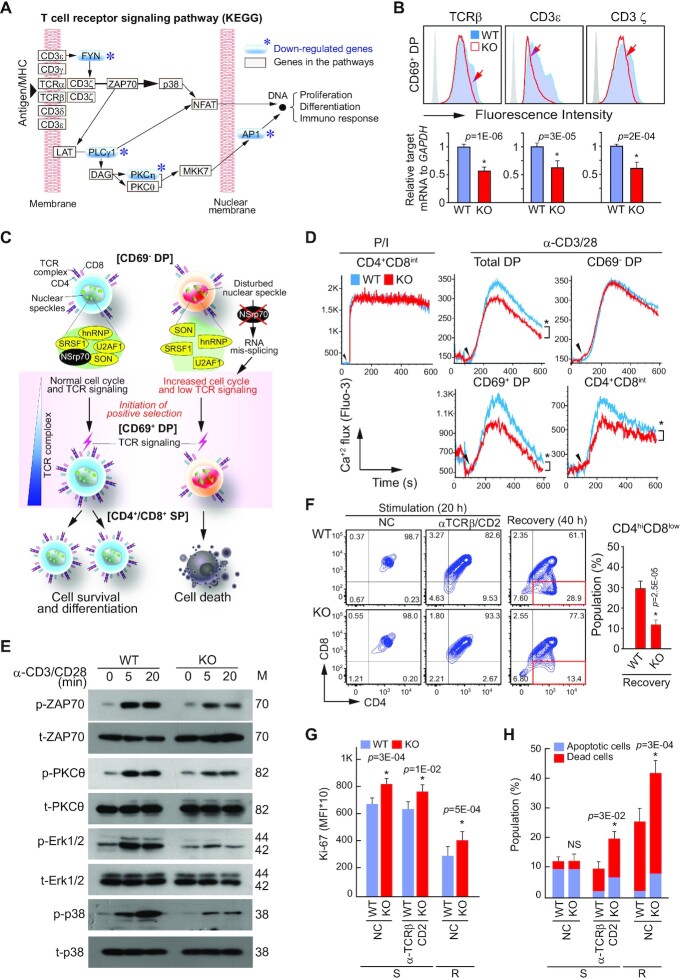
Deletion of NSrp70 results in defective survival signals following TCR activation in CD69^+^ DP thymocytes. (**A**) KEGG T cell receptor signaling pathway. The blue asterisks (*) represent down-regulated genes from the RNA-seq analysis (Figure 5B). (**B**) Expression of TCRβ, CD3ϵ, and CD3ζ on CD69^+^ DP thymocytes from *Nsrp1*^f/f^ (WT) and *Nsrp1*^f/f^CD4Cre (KO) mice. (**C**) A schematic model of gene regulation by NSrp70. NSrp70 sequesters splicing factors in the nuclear speckles. Disintegration of splicing factors by NSrp70 deletion induces abnormal gene regulation during thymocyte development. As one of the results, reduced TCR expression may cause impaired T cell maturation. (**D**) Calcium flux in DP thymocytes. Cells from (A) were stimulated with PMA and ionomycin (P/I) or anti-CD3/CD28 antibodies, and then calcium fluxes were measured by flow cytometry. (**E**) Western blot of ZAP70, PKCθ, Erk1/2, and p38 in DP cell lysates stimulated on anti-CD3/28 for 0, 5, and 20 min. β-actin served as the loading control. M, molecular mass (KDa). (**F**) *In vitro* thymocyte development assay. CD69^–^ DP thymocytes were stimulated on anti-TCRβ/CD2 antibodies for 20 h (stimulation), or the cells were further incubated for 20 h in medium without stimulation (recovery). (G and H) Cells from (F) were stained for Ki-67 (**G**) or annexin V and 7ADD (**H**). Cells were analyzed by flow cytometry (F–H). The bar graphs indicate mean fluorescence intensities (MFI) (G). *, meaningful *P*-value; NC, non-coated; S, stimulation; R, recovery. The bar graphs indicate average ± standard deviation of apoptotic and dead thymocytes population (H). NS, non-significant *P*-value. All data shown are representative of three independent experiments.

In the mRNA-seq analysis, we found that genes involved in the TCR signaling pathway are markedly downregulated, as depicted in the KEGG pathway (Figure 7A). A dramatic reduction of CD4^+^ or CD8^+^ SP thymocytes in NSrp70*-*cKO mice suggests that the survival signals following TCR activation may be impaired in DP thymocytes. As shown in Figure 4C, the spatiotemporal organization of pre-mRNA splicing factors and their normal protein isoforms may be critical to optimally express molecules required for T cell differentiation. An NSrp70 defect can induce unbalanced, polarized expression of certain protein isotypes, which may be coupled with the low expression of survival factors such as Bcl-2, p21, and Mcl-1 (Figure 6D). Interestingly, the expression of TCRβ, CD3ϵ, and CD3 ζ-chain was significantly reduced in NSrp70-cKO CD69^+^ DP thymocytes (Figure 7B). Attenuated TCR signaling, which is essential for thymocyte survival and differentiation, may result in retarded differentiation of SP thymocytes by coupling enhanced cell growth and reduced cell survival in NSrp70-deficient thymocytes, as schematically outlined in Figure 7C. To test this concept, we determined the Ca^2+^ flux induced by anti-CD3/CD28 or phorbol ester/ionomycin (P/I). Although the Ca^2+^ flux evoked by P/I was similar in both cKO and WT cells, the Ca^2+^ flux evoked by anti-CD3/CD28 was significantly reduced in NSrp70-cKO CD69^+^ DP thymocytes, but not in CD69^–^ DC thymocytes (Figure 7D). Accordingly, TCR down-stream signals were clearly attenuated (Figure 7E).

After the initiation of positive selection, DP thymocytes down-modulate CD4 and CD8 expression on the surface, entering the CD4^+^CD8^int^ transitional stage before committing to the CD4^+^ or CD8^+^ SP lineage (35,36). Therefore, we performed a two-stage differentiation assay *in vitro* (25) and found that only small numbers of NSrp70-cKO DP thymocytes were entering into the CD4^+^CD8^int^ stage. As a result, the populations of CD4^+^CD8^int^ were significantly lower in NSrp70-cKO cells than in WT cells (Figure 7F). Similar to the *in vivo* condition, NSrp70-cKO CD69^+^ DP thymocytes showed a higher proliferative phenotype (Ki-67^+^ cells) than that of WT cells (Figure 7G). In addition to the proliferative phenotype, these cells underwent increased apoptotic cell death, presumably due to the reduced expression of survival factors (Figure 7H).

Since rapid proliferation is coupled with impaired SP maturation in NSrp70-deficient CD69^+^ DP thymocytes, we next questioned whether blocking of the cell cycle may overcome the developmental defects. To determine this, NSrp70-cKO DP thymocytes were treated with the CDK1 inhibitor purvalanol A and a two-stage differentiation assay was performed. We found that the NSrp70-cKO DP thymocytes treated with purvalanol A did not differentiated into CD4^+^CD8^low^ intermediate thymocytes as shown in WT cells (Supplementary Figure S5A). However, purvalanol A significantly blocked apoptotic cell death (Supplementary Figure S5B), suggesting that uncontrolled proliferation is, to some extent, connected with SP development.

### NSrp70 deficiency results in severe peripheral lymphopenia

**Figure 8.**
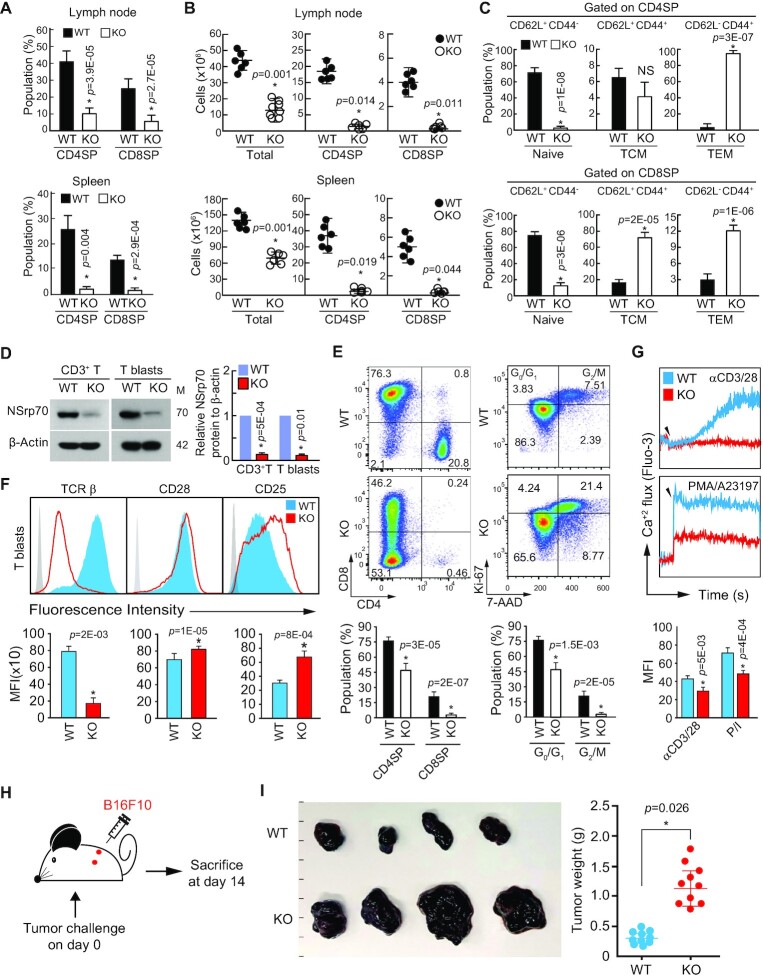
NSrp70 deficiency results in severe peripheral lymphopenia. (**A**, **B**) Flow cytometric analysis of CD4 and CD8 on T cells from lymph node (top) and spleen (bottom) of WT and KO mice. Quantification of CD4^+^ and CD8^+^ populations (A) and cell numbers (B) were represented as bar graphs (A) and circle symbols (B). Small horizontal lines indicate the mean ± standard deviation. Each black and white circle represents an individual mouse. *, meaningful *P*-value. (**C**) Analysis of CD62L and CD44 on T cells from (A). Numbers in quadrants indicate percent cells in each throughout. (**D**) Western blot analysis of NSrp70 in CD3^+^ T cells (left) and anti-CD3/28 and IL-2-induced CD3^+^ T blasts (right) obtained from WT and KO lymph nodes. M: molecular mass (KDa). β-actin served as a loading control. (**E** and **F**) Flow cytometric analysis of CD4^+^ and CD8^+^ and Ki-67^+^ and 7-ADD^+^ (E) and TCRβ, CD28, and CD25 (F) on CD3^+^ T blasts from (D). Numbers in the areas indicate percent cells in each throughout. (**G**) Calcium flux of CD3^+^ T blasts from (D). Calcium flux was measured as described in Figure 6D. (**H**) Schematic experimental design of B16F10 tumor model. (**I**) B16F10 cells were subcutaneously injected into left and right flank of WT and KO mice. Mice were sacrificed after 2 weeks. Tumor sizes and weights were determined. All data shown are representative of three independent experiments. *, meaningful *P*-value.

Finally, we examined the T cell compartment in the periphery of *Nsrp1*^f/f^ CD4Cre mice. Severe defects in CD4^+^ or CD8^+^ SP thymocytes in the thymus suggested the reduction of CD4^+^ or CD8^+^ populations in the periphery (Figure 2). As expected, the number of naive CD4^+^ T and CD8^+^ T cells was greatly reduced in both lymph nodes and spleens (Figure 8A and B). However, peripheral CD4^+^ T and CD8^+^ T cell populations from NSrp70-cKO mice had a greater frequency of memory (CD44^hi^CD62L^lo^ or CD44^hi^CD62L^hi^) cells (Figure 8C), a frequent observation in partially lymphopenic mice that reflects homeostatic expansion of T cell populations in the periphery (37). We further confirmed the deficiency of NSrp70 in the T cells isolated from peripheral lymphoid organs (Figure 8D). After stimulation with anti-CD3/28 and IL-2 for induction of T cell blasts, NSrp70-deficient CD3^+^ T cells showed abnormal phenotype of T cell blasts, with reduced population of CD8^+^ T blasts and very low numbers of CD4^+^ T blasts (Figure 8E). However, T blasts contained excessive Ki-67^+^ populations as was seen in the thymus (Figure 8E). The expression of TCRβ was down-modulated (Figure 8F) and Ca^2+^ flux driven by anti-CD3/28 or P/I was dramatically impaired (Figure 8G). However, upregulation of the IL-2 receptor CD25 may correspond with increased proliferation (Figure 8F). The increased expression of CD25 in NSrp70-cKO T blasts led us to investigate the population of CD4^+^Foxp3^+^ T regulatory cells in the spleen and thymus. However, no significant changes of the T regulatory cells and their populations were observed in NSrp70-cKO mice (Supplementary Figure S6).

To evaluate the pathological relevance of NSrp70 defects in mice, we examined cancer growth after subcutaneous injection of B16/F10 melanoma cells (Figure 8H). B16/F10 cells injected into *Nsrp1*^f/f^ CD4Cre mice developed tumor volumes and weights more than twice as much as observed in WT mice (Figure 8I). Collectively, the results of these experiments demonstrated that NSrp70 is a critical regulator of T cell development that corresponds with the regulation of T cell proliferation and survival mainly via the regulation of alternative splicing and spatiotemporal organization of pre-mRNA splicing factors and spliceosome-related factors located in nuclear speckles.

## DISCUSSION

Early development is characterized by a very high rate of proliferation of embryonic cells, which then differentiate to produce many specialized types of cells for tissues and organs (38,39). NSrp70 is expressed in very early embryonic stages and deletion of NSrp70 results in embryonic lethality, predicting that NSrp70 is essential for proliferation and development of cells during embryogenesis. In the present study, we found that NSrp70 is highly expressed in the lymphocyte-lineage and deletion of NSrp70 results in uncontrolled cell proliferation and cell death, suggesting that this protein is a lymphocyte essential factor that controls cell cycle and cell survival. Interestingly, NSrp70-deficiency resulted in remarkable changes in AS events of other pre-mRNA splicing factors, such as *Srsf1, Srsf7*, and *Son*, which are important for the cell cycle progression and oncogenic cellular transformation (16,17,40), and spliceosome-related factors, including *Sf3b1* and *U2af2* (18). In addition, this event was associated with increased expression of cell cycle regulatory genes and downregulation of genes involved in TCR signal transduction. As a result, mice showed impairment of T cell development and severe lymphopenia in the periphery.

In addition to prior *in silico* analysis (3), western blot analysis in the present study revealed that NSrp70 is highly and selectively expressed in lymphocytes. This suggests that NSrp70 has a lineage-specific role, especially in lymphocyte immunity. Consistent with this, our unpublished results show that deletion of NSrp70 in pre-B cells results in a severe defect of B cell development, followed by B cell lymphopenia. In contrast, we found that NSrp70 deletion in the developmental neurons resulted in no sign of defects in young mice. Since pre-mRNA splicing factors including snRNPs and many non-snRNPs are important for embryogenesis, knockouts of some individual genes in mice are known to induce early embryonic lethality (14). However, little is known of the alternative splicing factor that is selectively expressed and plays a critical role in lymphocytes. Two prototype SR-proteins, SRSF1 and SRSF2, have been reported to cause defects in B cell survival and T cell maturation (12,41), but they are not specific in lymphocytes. Interestingly, however, they are associated with NSrp70 by binding to the RS-like region, and exert an opposite splicing activity to target genes *in vitro* (19). Moreover, they have an opposite function for EO771 cancer cell transformation in the current study. This suggests that their roles are not independent, but probably affect the action mutually *in vivo*.

Interestingly, the number of isoforms and genes were markedly reduced in DP thymocytes from NSrp70-cKO mice, implying remarkable changes in the alternative splicing landscape. In agreement with this, the deletion of NSrp70 affected the AS events of genes involved in mRNA processing and splicing, DNA repair, and T cell differentiation pathways, some of which―*Srsf1, Srsf7, Son* (16,17,28), and etc.―physically interact with NSrp70. Thus, the current results strongly suggest that NSrp70 plays a pivotal role of AS events in the developmental process and that NSrp70 potentially regulates T cell development through multiple pathways including cell cycle, translation, and phosphorylation. The current results also indicate that gene clusters that control pre-mRNA splicing may regulate their expressions mutually. Through this mechanism, NSrp70 may counteract the function of other physically associating splicing factors. For instance, the splicing factor SRSF1 is also known as an oncoprotein (40,42). Overexpression of SRSF1 promotes the expression of isoforms that stimulate cell proliferation (40). In contrast, the present study showed that rapid cell proliferation was achieved by NSrp70 deletion, suggesting a contrasting role in cell cycle regulation. In agreement with this, analysis of genetic alterations in the human *NSRP1* gene in patients with different cancer types using TCGA PanCancer Atlas database and the cBioPortal online tool (www.cbioportal.org) demonstrated that *NSRP1* was altered in 178 samples from 10,953 patients, and was frequently amplified in several different cancer types (Supplementary Figure S7A). Using the ‘survival’ tab with the Kaplan-Meier plot and log-rank test, we found that *NSRP1* amplification was associated with a high overall survival rate in the affected patients compared with that in patients without *NSRP1* alteration (log-rank *P* = 0.0281) (Supplementary Figure S7B). These results suggest that NSrp70 can act to suppress severe cancer progression and growth. This function may also be linked with the role of NSrp70 in sequestering the pre-mRNA splicing factors in nuclear speckles. Alteration of NSrp70 expression may thus control the activity of other pre-mRNA splicing factors by regulating their expression as well as activity, thereby globally regulating the expression of downstream genes and their alternatively spliced forms. Further studies are now underway to determine whether NSrp70 acts as a tumor suppressor.

Pre-mRNA splicing occurs in 97% of human genes. This means that not all cellular physiological pathways, but most of them rely on the splicing machinery. This raises the intriguing question of how NSrp70 regulates cell cycle progression of DP thymocytes. As described earlier, alternative splicing regulation of cell cycle-related splicing factors, such as *Srsf1, Srsf7*, and Son, by NSrp70 can lead to a pivotal role to control cell cycle during thymocyte development (16,17,28). However, several recent studies indicate that AS events are also closely related to the cell cycle and cancer development (18,43,44). For instance, pre-mRNA splicing of key cell cycle regulators, such as CDC25, ARUKB, CLK1 and CDK2, can affect cell proliferation at the stage of mitotic division or next cell cycle transition (18). It has been shown that many of the AS events are controlled by the SR protein kinase CLK1 (44). CLK1 disruption leads to multiple cell cycle defects and loss of proliferation, whereas the opposite is associated with a variety of cancers (44). In the present study, interestingly, we found that *Clk1* is also a target gene of AS in NSrp70-cKO DP thymocytes (Supplementary Table S2). Another possibility is abnormal RNA splicing, typified by widespread RI. RI is common throughout cancers even in the absence of mutations which directly affect the RNA splicing machinery (45). It is well noticed that almost all liquid and solid cancer types exhibit frequent retention of both alternative and constitutive introns relative to control normal tissues (45). Since the removal of intron sequence from transcripts is catalyzed by the spliceosome (U1, U2, U4, U5 and U6), a multicomponent complex that assembles on the nascent pre-mRNA, a strong correlation between increased RI and decreased expression of U1 and U2 subunits already noticed (46). Therefore, we claim that alternative splicing of *Sf3b1* and *U2af2*, spliceosome sub-components, as revealed in the current study, is important for cell cycle progression and proliferation of NSrp70-cKO DP thymocytes.

Although numerous genes are involved in the maturation process of DP thymocytes to SP, the actions of those genes can be categorized around two critical control points. One is the genes for cell cycle and cell viability. We observed that active proliferation was accompanied by increased apoptosis in NSrp70-cKO thymocytes *in vivo* and *in vitro*. This raises the question of how increased cell proliferation is coupled with cell death. Perhaps the proteins that function in cell cycle pathways may also act to sensitize cells to apoptotic cell death. Indeed, uncontrolled cell proliferation may result in pathologic conditions, such as neoplasia, if it is not countered by appropriate cell death (47). Thus, our current results demonstrate that NSrp70 is a crucial factor in regulating the cell cycle and cell death, thereby maintaining homeostasis during T cell development. Uncontrolled cell division and apoptosis at the DP stage, during which cells need differentiation and selection instead of cell division, may impede further progress of development to the SP stage. The second critical control point is the productive rearrangement of TCRβ and TCRα, which is critical for positive and negative T cell selection (48). Significant reduction of TCRβ^hi^ population in CD69^+^ DP thymocytes demonstrates that TCR signaling is not optimal in NSrp70-cKO CD69^+^ DP thymocytes, thereby leading to a severe defect in SP maturation. Uncontrolled cell proliferation and apoptosis may reduce the number of cells that properly express surface TCR complex in CD69^+^ DP thymocytes. Furthermore, the decreased expression of TCR downstream signaling molecules will lead to a lack of survival signaling after TCR activation, which may be eventually associated with the death of SP thymocytes. However, dramatic elimination of NSrp70-depleted cells may suggest another factor is necessarily required for T cell maturation. In this point of view, abnormal expression of TCR complex is also occurred by the stage-specific transcription factors during positive selection (11,49–53). However, the absence of expression alterations of the transcription factor suggests NSrp70 is not involved in the regulation of transcription factors for SP maturation.

Despite a severe block at the transition from DP to SP thymocytes, a fraction of NSrp70-deleted cells was able to reach the periphery. This suggests that these T cells may express proper quantities of chemokine receptors on their cell surface. However, because they do not express TCRβ properly, these T cells are unlikely to function as normal T cells. In addition, it was interesting to note that these peripheral T cells still proliferate rapidly as compared to that of WT T cells. This fact further supports the idea that NSrp70 directly controls the cell cycle, and in turn this mechanism influences the development of DP thymocytes to SP. Overexpression of CD25, a receptor for IL-2, may also reflect the rapid proliferation as seen in NSrp70-deleted DP thymocytes. However, severe lymphopenia in the periphery resulted in tumor control failure.

In conclusion, the present study establishes an important role for the novel splicing factor NSrp70 in T cell development, especially at the transition between DP and SP stages. To our knowledge, NSrp70 is the first splicing regulator predominantly expressed in lymphoid-lineage cells. However, NSrp70 is also highly expressed in CD4^+^ and CD8^+^ matured SP T cells. Thus, it will be interesting to determine whether NSrp70 also controls differentiation of CD4^+^ or CD8^+^ naive T cells to effector or memory T cells, by breeding floxed *Nsrp1* mice with variety stage-specific Cre-transgenic mice. It is also largely unknown whether NSrp70 affects the development of regulatory T cells or other subsets of T cells. By single cell analysis, we are deciphering how NSrp70 globally affects the fate decision of lymphocytes.

## DATA AVAILABILITY

The sequencing data were deposited into GEO repository with the accession number GSE168379.

## Supplementary Material

gkab389_Supplemental_Files

## Notes

Present address: Young-Dae Kim, Institute for Drug Discovery, CrystalGenomics, Inc., Korea Bio Park, 700 Daewangpangyo-ro, Bundang-gu, Seongnam-si, Gyeonggi-do 13488, Korea.
