# Supplementary material for: NSrp70 is a lymphocyte-essential splicing factor that controls thymocyte development
Source: Nucleic Acids Res. 2021 May 25;49(10):5760–78. doi: 10.1093/nar/gkab389 (PMC8191771; doi:10.1093/nar/gkab389)
Supplement: gkab389_Supplemental_Files [file gkab389_supplemental_files.zip › Figures S1~S7.pdf]

Figure S1. Schematic diagram of *Nsrp1* conditional knockout and the resulting *Nsrp1*$^{f/f}$ CD4Cre mouse

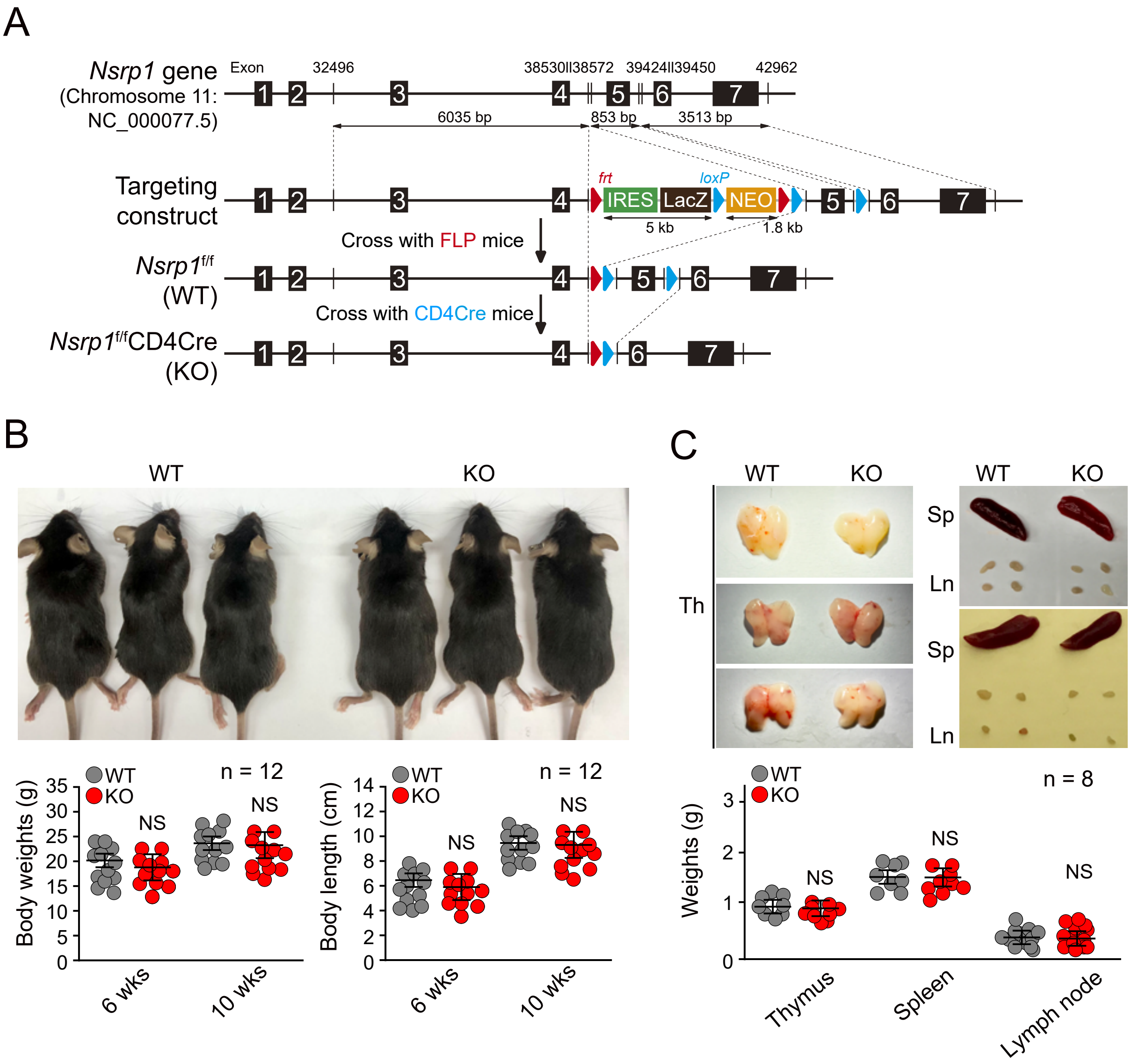

Figure S2. *Nsrp1*-deficiency results in reduced expression of maturation markers and reveals defect of differentiation into the SP thymocytes

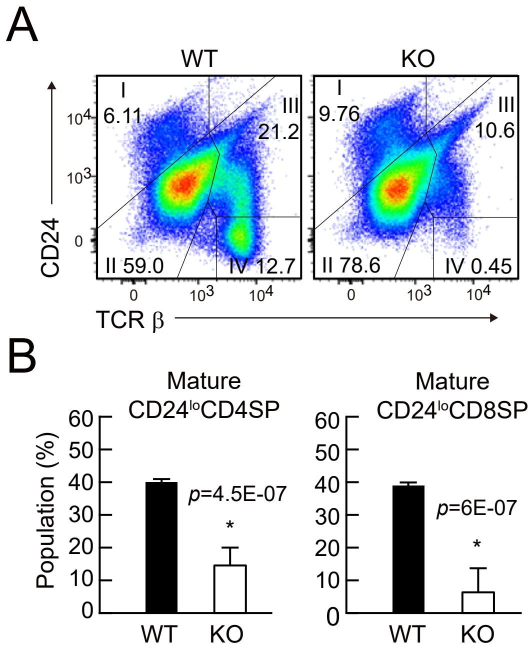

Figure S3. NSrp70 physically interacts with RNA splicing regulators and is important for speckle organization

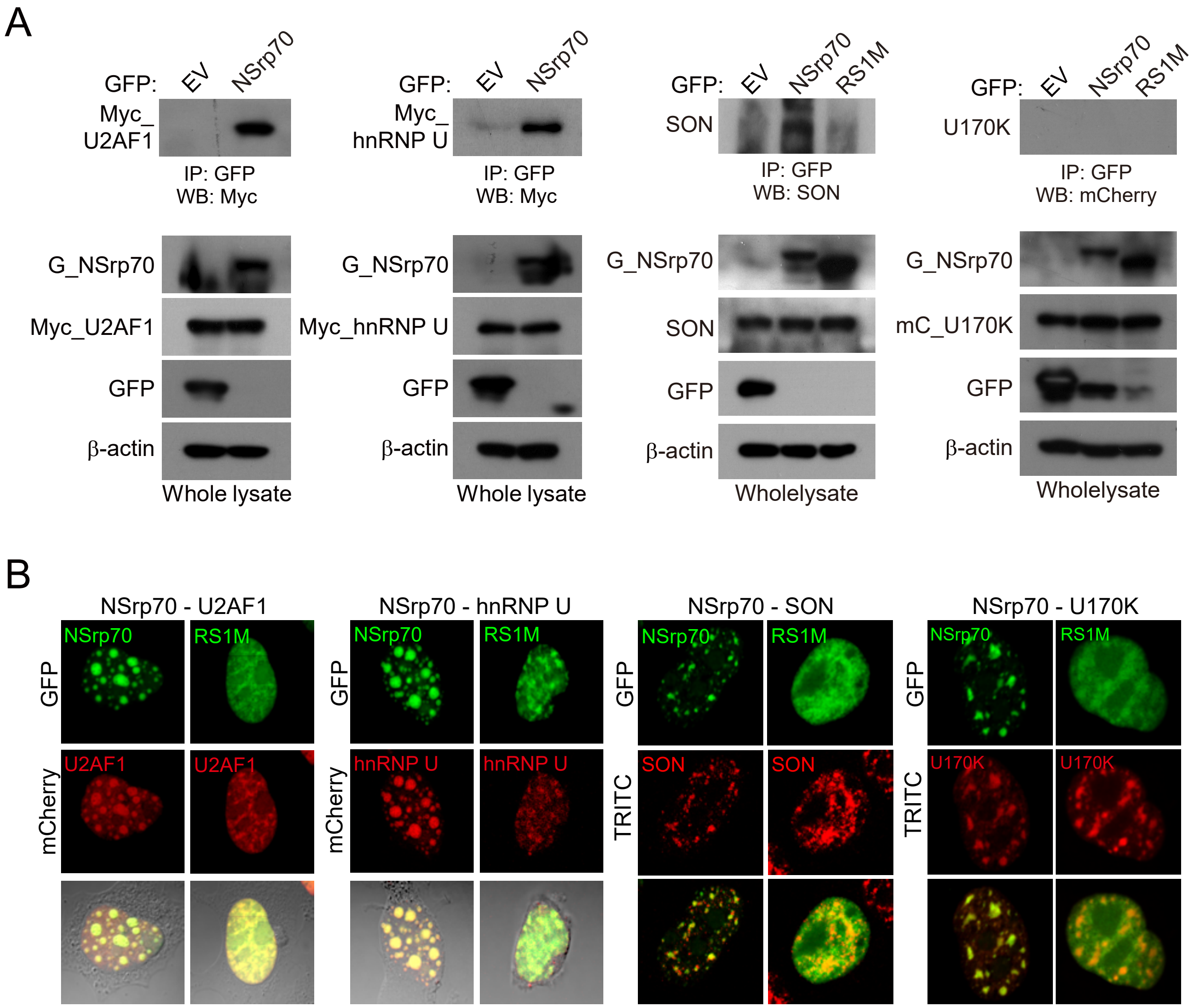

Figure S4. NSrp70-deficiency increases the expression of cell cycle regulators, but not the genes in thymocyte development

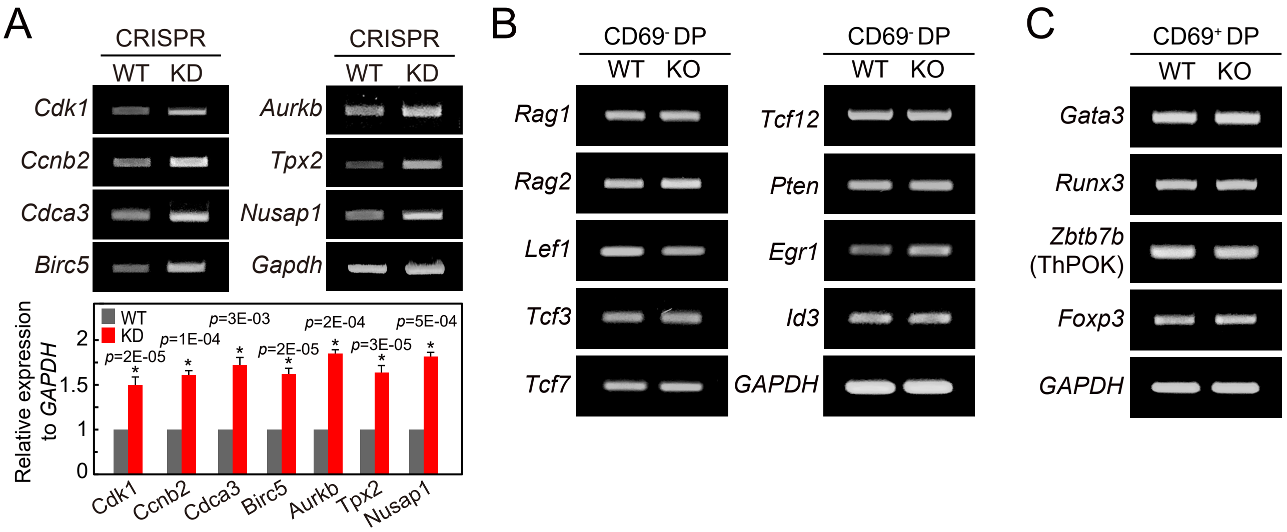

Figure S5. Inhibition of cell cycle regulator blocks apoptotic cell death in *Nsrp1*-deficient DP thymocytes

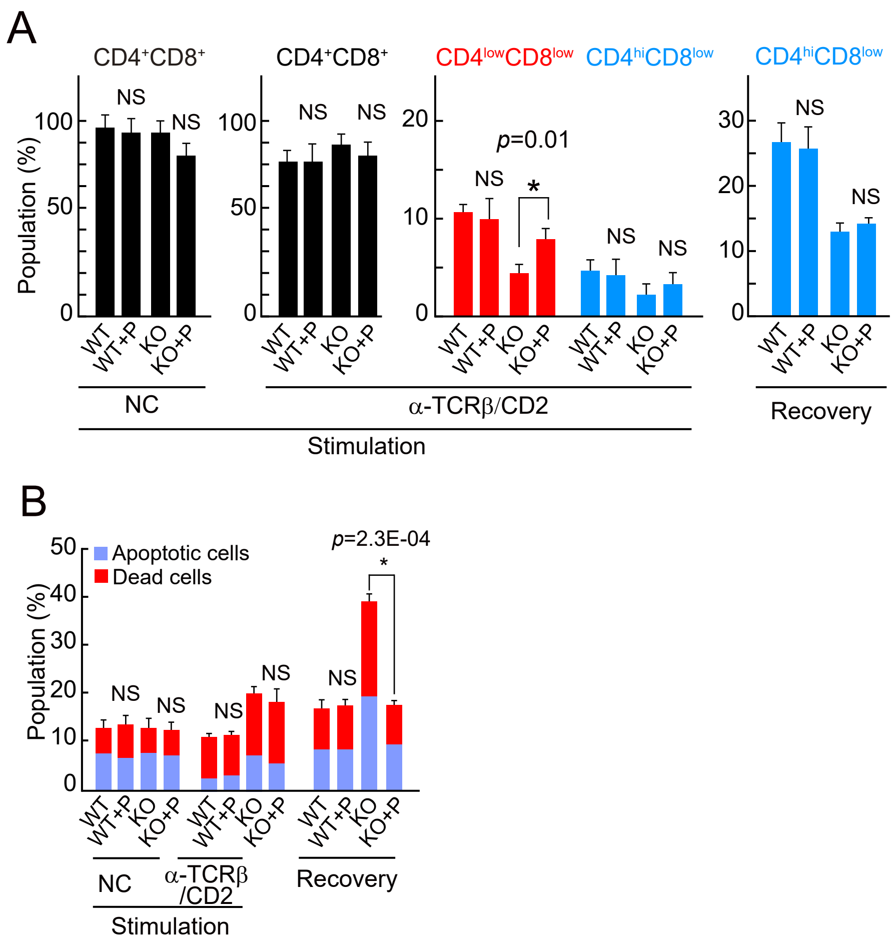

Figure S6. NSrp70 deficiency does not significantly change the population of regulatory T cells

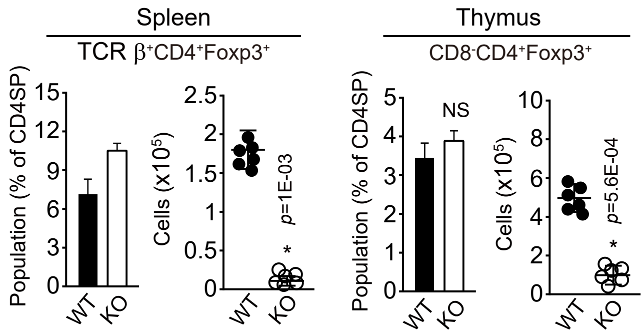

Figure S7. Amplification of the *NSRP1* gene in tumor increases the survival rate of cancer patients.

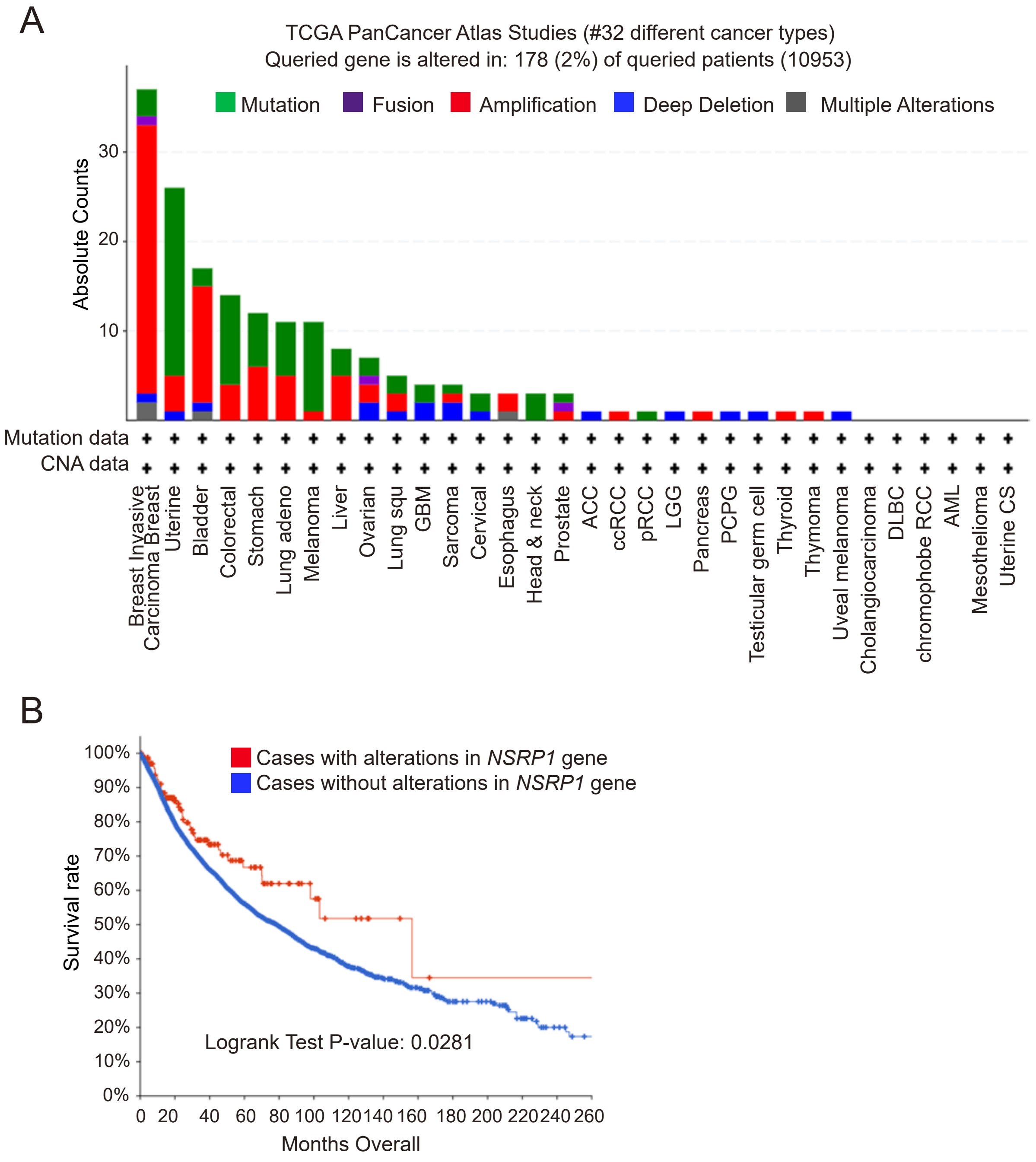

	Altered group	Unaltered group
Number of Cases, Total	175	10626
Number of Casese, Deceased	44	3470
Median Months Overall	156.49	78.44
