# Supplementary material for: NSrp70 is a lymphocyte-essential splicing factor that controls thymocyte development
Source: Nucleic Acids Res. 2021 May 25;49(10):5760–78. doi: 10.1093/nar/gkab389 (PMC8191771; doi:10.1093/nar/gkab389)
Supplement: gkab389_Supplemental_Files [file gkab389_supplemental_files.zip › Revised supplementary information.docx]

# SUPPLEMENTARY INFORMATION

# Supplementary figures

**Supplementary Figure 1. Schematic diagram of *Nsrp1* conditional-knockout and the resulting *Nsrp1*^f/f^CD4Cre mouse.** (A) Targeting strategy to avoid gene interference and genetic ambiguity caused by the presence of the neomycin selection marker in the *Nsrp1-*floxed allele. *Nsrp1*^+/flox-frt-neo^ mice were crossed with FLP transgenic mice to generate neo-free *Nsrp1*^+/flox^ mice. Next, *Nsrp1*^+/flox^ mice were crossed with CD4Cre transgenic mice to facilitate *Nsrp1* gene deletion in late DN thymocytes under the control of the CD4 enhancer/promoter elements. f/f, flox/flox. (B and C) Phenotypic comparison of *Nsrp1*^f/f^ (WT) and *Nsrp1*^f/f^CD4Cre (cKO) mice. (B) Body weights of *Nsrp1*^f/f^ (WT) and *Nsrp1*^f/f^CD4Cre (KO) mice at 6- and 10-weeks after birth. Data represent mean ± standard error of the mean (n = 8). NS, non-significant p-value. (C) Weight changes of organs from 6-week-old *Nsrp1*^f/f^ (WT) and *Nsrp1*^f/f^CD4Cre (KO) mice. Th, Thymus; Sp, Spleen; Ln, Lymph node; NS, Non-significant p-value. All data shown are representative of three independent experiments.

**Supplementary Figure 2. *Nsrp1*-deficiency results in reduced expression of maturation markers and reveals defect in differentiation into SP thymocytes.** (A) Flow cytometric analysis of CD24 and TCRβ gated on DN thymocytes from Figure 2D (left). (B) Quantification of average percent of mature CD24^lo^SP thymocytes. Bar graphs indicate the mean ± standard deviation of three independent experiments. *, meaningful p-value.

**Supplementary Figure 3. NSrp70 physically interacts with RNA splicing regulators and is important for speckle organization.** (A) Immunoprecipitation and immunofluorescence analysis of NSrp70 and other splicing factors. HEK293T cells were transfected with GFP (EV) or GFP_NSrp70 (G_NSrp70). Samples were immunoprecipitated and blotted with antibodies against the indicated proteins. IP, immunoprecipitation; WB, western blotting. (B) HEK293T cells were co-transfected with G_NSrp70 or its mutant (G_RS1M) and indicated proteins which were fused with mCherry (U2AF1, hnRNP U, and U1170K) or endogenous protein (SON). Fluorescence signals were visualized under a confocal microscope. Magnification, 100 ×. Results are representative of three independent experiments.

**Supplementary Figure 4. NSrp70-deficiency increases the expression of cell cycle regulators, but not the genes in thymocyte development.** (A) RT-PCR analysis for the potential NSrp70-controled cell cycle target candidates in HEK293T cells. HEK293T were transfected with all-in-one CRISPR/Cas9 vector (GFP) to knock down NSrp70. GFP^+^ cells were sorted, and the cell lysates were subjected to western blot analysis. (top) mRNA levels of indicated targets were assessed by RT-PCR. *Gapdh* served as a loading control. (bottom) Bar graphs indicate average ± standard deviation of three independent experiments. (B and C) RT-PCR analysis of thymocyte development-related targets (B) and thymocyte lineage commitment-related targets (C) in sorted CD69^-^ or CD69^+^ DP thymocytes. mRNA levels of indicated targets were assessed by RT-PCR. *Gapdh* shown as the loading control. All data shown are representative of three independent experiments.

**Supplementary Figure 5. Inhibition of cell cycle regulator blocks apoptotic cell death in *Nsrp1*-deficient DP thymocytes.** (A) *In vitro* thymocyte development assay with treatment of cell cycle inhibitor. CD69^-^ DP thymocytes were sorted and incubated with the cell cycle inhibitor purvalanol A (200 nM) or DMSO as a negative control. Sorted thymocytes were stimulated by plate-coated anti-TCRβ/CD2 antibodies for 20 h, and immediately analyzed by flow cytometry (stimulation), or the cells were further washed, incubated for an additional 20 h in medium without stimulation, and analyzed by flow cytometry (recovery). *, meaningful p-value; NC, non-coated; P, purvalanol A. (B) Cells from (A) were stained for annexin V and 7ADD, and then analyzed by flow cytometry. The bar graphs indicate the mean ± standard deviation of apoptotic and dead thymocyte populations performed three times.

**Supplementary Figure 6. NSrp70 deficiency does not significantly change the population of regulatory T cells.** Flow cytometric analysis of TCRβ and Foxp3 gated on CD4^+^ T cell in spleen and thymus from *Nsrp1*^f/f^ (WT) and *Nsrp1*^f/f^CD4Cre (KO) mice (left). Quantification of average percent of T regulatory cells (right). Bar and dots graphs indicate the mean ± standard deviation of three independent experiments. *, meaningful p-value.

**Supplementary Figure 7. Amplification of human *NSRP1* gene in tumors increases the survival rate of cancer patients.** (A) Alteration of human *NSRP1* gene in 32 different cancer types was analyzed in TCGA PanCancer Atlas dataset using the cBioPortal online tool. The absolute counts of gene alteration in each dataset are shown in different color bars. (B) Overall survival rates among cancer patients with or without *NSRP1* mutation were compared using Kaplan-Meier plots and Logrank tests.

**Supplementary Table 1. Primer sequences used for qRT-PCR analysis.**

**Supplementary Table 2. List of alternatively spliced-genes affected by NSrp70 knockout based on MISO analysis (ΙΔΨΙ ≥ 0.2, BF ≥ 10).**

**Supplementary Table 3. Statistics of differentially expressed genes (DEG).** We identified significantly differentially expressed genes by negative binomial tests of DESeq2 R package (adjusted p-value < 0.05). From differential gene expression analysis, we estimated the average gene expressions (normalized gene counts), log2 fold change, standard errors of log2 fold change, p-value of negative binomial tests, and adjusted p-value.

**Supplementary Table 4.** Significantly enriched gene ontology biological process terms from up- or down-regulated genes (p-value < 0.01). We showed significantly enriched biological process terms (i.e. GO BP DIRECT) with p-values and type of enrichment (up or down-regulation).

**Supplementary Table 5.** Significantly enriched gene ontology cellular component (i.e. subcellular location) terms from up- or down-regulated genes (p-value < 0.01). We showed significantly enriched cellular component terms (i.e. GO CC DIRECT) with p-values and type of enrichment (up or down-regulation).
